# Supplementary material for: Amyloidogenic proteins in the SARS-CoV and SARS-CoV-2 proteomes
Source: Nat Commun. 2023 Feb 20;14:945. doi: 10.1038/s41467-023-36234-4 (PMC9940680; doi:10.1038/s41467-023-36234-4)
Supplement: Supplementary file 1 — Supplementary Information [file 41467_2023_36234_MOESM1_ESM.pdf]

Supplementary Information

Amyloidogenic proteins in the SARS-CoV & SARS-CoV-2 proteomes

Taniya Bhardwaj[1], Kundlik Gadhave[1], Shivani K Kapuganti[1], Prateek Kumar[1], Zacharias Faidon Brotzakis[2], Kumar Udit Saumya[1], Namyashree Nayak[1], Ankur Kumar[1], Richa Joshi[1], Bodhidipra Mukherjee[1], Aparna Bhardwaj[1], Krishan Gopal Thakur[3], Neha Garg[4], Michele Vendruscolo[2], Rajanish Giri[1]*

[1]School of Biosciences and Bioengineering, Indian Institute of Technology Mandi, Kamand, Himachal Pradesh, 175075, India.

[2]Centre for Misfolding Diseases, Yusuf Hamied Department of Chemistry, University of Cambridge, Cambridge CB2 1EW, UK

[3]Structural Biology Laboratory, G. N. Ramachandran Protein Centre, CSIR-Institute of Microbial Technology, Chandigarh, 160036, India.

[4]Department of Medicinal Chemistry, Faculty of Ayurveda, Institute of Medical Sciences, Banaras Hindu University, Varanasi, Uttar Pradesh, 221005, India.

Supplementary tables:

Supplementary table 1: Distribution of APRs in SARS-CoV-2 structural proteins.

SARS-CoV-2 protein (length in amino acids) (NCBI/Uniprot accession ID)	MetAmyl (% of APRs)	FISH Amyloid (% of APRs)	AGGRESCAN (% of APRs)	FoldAmyloid (% of APRs)	CamSol (% of hydrophobicity)
**Spike glycoprotein** (1273) (YP_009724390.1)	1-20, 28-33, 42-52, 58-73, 88-97, 105-110, 114-121, 124-132, 139-145, 166-176, 192-205, 226-236, 262-272,	35-39, 54-58, 89-93, 116-120, 125-129, 139-146, 167-171, 179-183, 200-204,	1-8, 10-16, 31-38, 40-47, 50-56, 58-70, 86-91, 100-109, 114-133, 140-145, 162-167, 189-208, 231-247, 260-271,	1-10, 34-38, 53-69, 100-108, 116-120, 126-135, 141-146, 174-178, 190-195, 235-244,	1-10, 55-68, 117-121, 125-134, 263-269, 431-436, 487-492, 508- 516, 540-544, 592-598, 608-613, 692-698, 717-

	284-289, 302-308, 311-336, 347-352, 357-384, 390-409, 428-437, 443-448, 472-477, 495-500, 507-517, 523-528, 534-544, 546-554, 583-600, 605-622, 635-645, 651-660, 670-675, 687-697, 703-708, 717-727, 729-744, 756-763, 767-772, 776-786, 801-806, 822-830, 855-861, 869-875, 879-885, 906-918, 939-944, 947-952, 958-963, 969-981, 991-998, 1003-1013, 1029-1037, 1056-1071, 1075-1081, 1092-1107, 1119-1137, 1159-1164, 1171-1181,	266-270, 325-329, 365-370, 373-377, 399-403, 406-410, 446-450, 469-473, 482-486, 502-506, 540-544, 550-554, 558-562, 591-599, 609-613, 689-693, 783-789, 822-826, 914-918, 953-957, 1001-1005, 1059-1068, 1127-1132, 1175-1180, 1206-1210, 1228-1232 **(15.47%)**	303-311, 331-336, 338-354, 362-383, 390-400, 428-437, 448-457, 484-490, 508-520, 584-588, 590-599, 610-617, 666-671, 688-698, 716-727, 729-745, 751-759, 761-768, 783-787, 797-805, 817-833, 853-861, 864-870, 872-896, 898-914, 961-982, 1000-1013, 1044-1052, 1058-1068, 1097-1101, 1124-1137, 1171-1179, 1207-1212, 1214-1249, 1266-1273 **(45.01%)**	264-269, 274-278, 327-331, 348-353, 366-370, 389-395, 431-436, 450-457, 487-492, 507-519, 539-543, 560-564, 582-586, 609-613, 691-697, 718-722, 738-743, 751-755, 780-784, 799-804, 819-825, 875-880, 894-907, 993-999, 1005-1014, 1047-1051, 1060-1067, 1101-1105, 1127-1132, 1210-1239 **(23.72%)**	724, 738-742, 752-761, 884-889, 895-899, 1004-1010, 1059- 1068, 1101-1106, 1127-1135, 1211-1242 **(15.16%)**

	1221-1255, 1236-1273 **(51.76%)**				
**Envelope** (75) (YP_009724392.1)	1-6, 9-63 **(81.3%)**	10-14, 16-23, 25-29, 55-59 **(30.66%)**	1-6, 11-53, 55-61 **(74.67%)**	11-51, 56-60 **(61.34%)**	12-34, 39-50, 54-59 **(54.6%)**
**Membrane** (222) (YP_009724393.1)	5-10, 21-40, 43-52, 60-82, 92-102, 118-123, 136-145, 167-173, 187-197, 208-213, 216-222 **(52.7%)**	25-29, 44-56, 69-73, 99-103, 108-112, 201-205 **(17.11%)**	19-38, 43-58, 60-108, 118-122, 124-131, 135-149, 171-181, 216-222 **(59%)**	18-39, 44-60, 62-77, 81-105, 108-112, 118-122, 126-132, 138-147, 176-180, 197-202, 218-222 **(57.65%)**	21-38, 46-79, 81-100 **(32.4%)**
**Nucleocapsid** (419) (YP_009724391.1)	12-17, 49-58, 108-113, 128-135, 154-162, 241-251, 265-275, 332-337, 346-355, 387-396, 412-417 **(22.19%)**	70-75, 159-163, 238-244, 348-352, 385-389, 405-409 **(7.87%)**	49-56, 107-116, 126-137, 156-161, 218-226, 310-322, 327-336, 348-356 **(18.37%)**	51-55, 85-89, 106-113, 130-134, 156-160, 219-226, 313-317, 329-333, 350-355, 392-396 **(13.6%)**	51-56, 107-113, 131-134, 156-160, 330-334 **(5.48%)**

Supplementary table 2: Distribution of APRs in SARS-CoV-2 accessory proteins.

**SARS-CoV-2 protein (length in**	**MetAmyl**	**FISH Amyloid**	**AGGRESCAN (% of APRs)**	**FoldAmyloid (% of APRs)**	**CamSol (% of APRs)**

amino acids) (NCBI accession ID)	(% of APRs)	(% of APRs)			
**ORF3a** (275) (YP_009724397.2)	6-17, 31-36, 43-67, 72-97, 109-125, 153-158, 160-172, 185-191, 196-207, 211-216, 220-237, 243-249, 251-260, 268-275, **(62.9%)**	44-53, 76-80, 86-90, 109-117, 119-125, 142-146, 166-170, 212-216, 224-233, 254-258 **(24%)**	1-14, 31-35, 37-41, 43-67, 73-103, 105-132, 141-158, 160-171, 197-218, 227-236 **(80%)**	1-10, 43-57, 77-97, 103-116, 118-133, 139-152, 154-159, 201-210, 212-217, 228-235, 244-248 **(45.45%)**	5-13, 42-57, 78-98, 106-118, 127-131, 143-151, 165-169, 202-206, 213-217, 227-234 **(27.63%)**
**ORF3b** (22) (P0DTF1)	8-22 **(68.18%)**	9-17 **(40.9%)**	4-22 **(86.36%)**	1-22 **(100%)**	2-22 **(95.4%)**
**ORF6** (61) (YP_009724394.1)	4-41 **(62.29%)**	7-11, 14-19, 28-37 **(34.4%)**	1-39 **(63.93%)**	1-10, 12-22, 24-38 **(59.01%)**	1-21, 24-35 **(54.09%)**
**ORF7a** (121) (YP_009724395.1**)**	3-15, 23-32, 35-40, 55-66, 69-77, 93-115 **(60.33%)**	5-9, 17-21, 100-111 **(18.18%)**	1-18, 26-33, 55-66, 100-116 **(45.45%)**	1-13, 16-22, 54-61, 73-77, 98-116 **(42.97%)**	1-13, 56-66, 99-114 **(33.05%)**
**ORF7b** (43) (YP_009725296.1)	2-12, 18-31 **(58.1%)**	12-28 **(39.53%)**	1-32 **(74.41%)**	1-32 **(74.41%)**	7-32 **(60.46%)**

**ORF8** (121) (YP_009724396.1)	1-17, 20-33, 37-42, 46-51, 57-67, 71-91, 95-105, 113-121 **(78.51%)**	2-10, 76-80, 107-111 (**15.7%)**	1-15, 39-49, 57-61, 71-84, 96-103, 114-121 **(50.41%)**	1-12, 39-49, 58-62, 81-88, 97-104, 113-121 **(43.8%)**	1-15, 78-88, 117-121 **(25.61%)**
**ORF9b** (97) (P0DTD2)	18-24, 30-35, 37-47, 71-80, 89-97 **(44.32%)**	40-45 (**6.18%)**	20-24, 44-50, 74-79, 90-97 **(26.8%)**	20-25, 42-48, 69-73, 91-95 **(23.71%)**	71-76, 92-97 **(12.37%)**
**ORF10** (38) (YP_009724396.1)	2-8, 11-18, 25-38 **(76.3%)**	23-27, 31-35 (**23.68%)**	1-9, 11-20, 25-38 **(86.84%)**	1-20, 31-37 **(71.05%)**	1-20, 32-38 **(63.15%)**
**ORF14** (73) (P0DTD3)	3-9, 27-42, 49-54, 65-73 **(52%)**	39-44 **(8.21%)**	34-39, 50-71 **(38.35%)**	29-38, 51-72 **(43.83%)**	2-7, 33-40, 59-72 **(38.35%)**

Supplementary table 3: Distribution of APRs in SARS-CoV-2 non-structural proteins.

**SARS-CoV-2 protein (length in amino acids) (NCBI accession ID)**	**MetAmyl (% of APRs)**	**FISH Amyloid (% of APRs)**	**AGGRESCAN (% of APRs)**	**FoldAmyloid (% of APRs)**	**CamSol (% of APRs)**
**NSP1** (180) (YP_009725297.1)	10-31, 50-61, 66-74, 84-89, 101-111, 114-119	59-66, 92-96, 105-114 **(12.77%)**	1-5, 20-31, 52-58, 68-72, 84-93, 103-108,	18-22, 25-29, 68-72, 83-88, 105-109, 116-124	15-20, 105-109 **(6.11%)**

	**(36.66%)**		110-114, 116-122 **(31.67%)**	**(19.44%)**	
**NSP2** (638) (YP_009725298.1)	46-53, 57-62, 94-105, 118-123, 134-139, 155-161, 182-193, 223-238, 248-263, 278-288, 292-313, 318-334, 339-344, 362-372, 378-401, 415-431, 433-442, 468-489, 492-503, 509-519, 525-535, 575-581, 598-608 **(44%)**	184-189, 257-261, 363-368, 424-428, 433-440, 458-462, 468-472, 494-498, 560-564 **(7.83%)**	78-82, 97-105, 118-124, 136-142, 158-162, 182-190, 225-241, 282-286, 291-315, 353-371, 382-445, 447-451, 463-487, 494-520, 524-528, 557-561, 571-576, 603-614, 625-632 **(42.31%)**	1-5, 48-52, 58-62, 92-96, 134-139, 186-191, 228-235, 241-245, 294-299, 362-370, 397-408, 415-421, 430-439, 460-464, 467-474, 496-501, 503-508, 603-612 **(19.43%)**	92-98, 123-128, 186-192, 226-235, 295-299, 351-355, 414-431, 434-439, 482-486, 496-500, 504-508, 602- 608 **(12.85%)**

**NSP3** (1945) (YP_009725299.1)	1-8, 10-17, 20-27, 39-48, 52-66, 102-108, 144-149, 166-175, 179-191, 198-212, 220-225, 234-242, 272-277, 279-291, 295-304, 331-336, 341-361, 367-374, 393-398, 417-429, 434-442, 451-462, 467-486, 504-512, 516-521, 526-545, 548-554, 570-585, 593-602, 604-620, 633-640, 652-670, 682-692, 702-707, 715-722, 730-737, 745-	10-14, 20-24, 201-205, 209-213, 218-222, 371-375, 379-383, 434-439, 452-456, 476-480, 504-508, 533-537, 580-584, 596-600, 716-720, 746-750, 755-760, 785-789, 823-827, 946-950, 964-968, 1010-1014, 1068-1072, 1106-1110, 1148-1153, 1253-1258, 1335-1339, 1396-1400, 1409-1413, 1423-1428, 1433-1437, 1456-1460, 1509-1513, 1518-1522, 1537-1541,	16-23, 42-46, 51-66, 68-73, 82-89, 98-106, 185-189, 201-219, 237-245, 248-252, 281-291, 295-301, 330-339, 341-365, 367-374, 417-421, 432-442, 453-458, 461-465, 468-483, 530-538, 550-558, 573-580, 582-586, 600-618, 632-637, 648-653, 667-672, 684-689, 735-740, 742-758, 763-771, 824-830, 843-848, 857-868, 891-903, 912-916, 928-935, 946-957, 986-991, 1029-1034, 1045-1050, 1062-1071, 1107-1112, 1137-1142, 1152-1157, 1172-1177, 1188-1199, 1252-	83-90, 100-106, 244-248, 294-299, 324-329, 342-349, 353-359, 435-440, 479-483, 506-510, 534-540, 553-562, 602-608, 614-619, 632-637, 646-650, 694-698, 707-711, 748-755, 799-803, 814-818, 825-830, 856-866, 879-884, 891-899, 915-919, 930-935, 959-954, 1108-1115, 1139-1143, 1172-1176, 1189-1197, 1255-1259, 1276-1281, 1337-1345, 1351-1364, 1409-1428, 1433-1437, 1458-1463, 1488-1494, 1496-1505, 1507-1555, 1561-1582,	21-25, 41-45, 53-59, 101-106, 199-205, 239-244, 534-540, 551-555, 604-608, 629-638, 752-756, 762-771, 858-865, 892-899, 915-920, 931-935, 1191-1195, 1348-1363, 1410-1429, 1432-1437, 1458-1467, 1470-1474, 1489-1505, 1507-1554, 1564-1583, 1720-

	757, 759-768, 775-780, 783-789, 797-804, 808-813, 842-847, 863-868, 891-896, 899-908, 928-938, 942-950, 965-972, 975-981, 1002-1008, 1025-1031, 1045-1051, 1054-1060, 1062-1075, 1112-1117, 1136-1142, 1147-1155, 1171-1176, 1189-1194, 1206-1212, 1225-1230, 1243-1259, 1284-1289, 1319-1324, 1327-1343, 1346-1351, 1360-1367, 1380-1392, 1395-1400, 1404-	1547-1551, 1571-1577, 1615-1619, 1644-1648, 1720-1724, 1776-1780, 1813-1817, 1855-1859 **(11.41%)**	1259, 1282-1286, 1298-1315, 1327-1351, 1353-1364, 1383-1398, 1404-1441, 1455-1468, 1470-1479, 1485-1557, 1559-1585, 1604-1608, 1610-1624, 1636-1647, 1671-1678, 1720-1727, 1738-1749, 1751-1758, 1770-1786, 1788-1793, 1795-1799, 1807-1820, 1833-1838, 1856-1861, 1888-1900, 1927-1945 **(42.5%)**	1591-1595, 1614-1619, 1630-1635, 1670-1674, 1683-1688, 1701-1705, 1720-1725, 1741-1756, 1813-1817, 1835-1839, 1854-1859, 1891-1896 **(22.51%)**	1725, 1741-1746, 1777-1783, 1891-1895, 1933-1938 **(14.7%)**

	1437, 1443-1448, 1453-1477, 1486-1499, 1501-1525, 1529-1543, 1549-1556, 1563-1595, 1603-1613, 1615-1623, 1634-1648, 1668-1687, 1699-1706, 1718-1728, 1738-1746, 1758-1773, 1775-1787, 1791-1798, 1810-1819, 1821-1826, 1828-1837, 1846-1852, 1857-1862, 1880-1885, 1928-1941 **(51.61%)**				
**NSP4** (500) (YP_009725300.1)	9-35, 39-44, 48-54, 57-62, 90-105, 109-114, 120-137, 147-152, 154-161, 166-	16-24, 40-44, 52-56, 117-121, 128-133, 257-261, 288-293, 297-301, 336-	1-28, 41-49, 90-103, 114-122, 124-135, 137-157, 170-181, 193-201, 203-217, 236-240, 253-269, 275-	4-9, 11-29, 72-76, 89-97, 100-104, 118-127, 190-194, 211-215, 253-257, 262-271, 290-306, 312-	12-31, 90-94, 100-104, 119-124, 128-132, 253-258, 264-271, 286-

	171, 175-183, 208-218, 231-239, 253-266, 280-299, 311-320, 323-363, 373-389, 393-398, 402-412, 434-439, 444-449, 458-463, 483-488, 491-500 **(57.6%)**	347, 358-362, 403-412, 419-423, 444-448, 484-491 **(18.2%)**	306, 309-332, 334-338, 340-370, 375-409, 411-427, 430-436, 438-453, 494-500 **(65.8%)**	333, 342-354, 358-371, 373-385, 389-396, 398-405, 417-422, 426-430, 434-440, 442-449 **(40%)**	302, 314-352, 360-385, 387-396, 417-421, 495-500 **(31.6%)**
**NSP5** (306) (YP_009725301.1)	13-26, 33-47, 65-86, 89-94, 101-118, 123-128, 141-162, 166-176, 196-215, 223-228, 246-251, 254-262, 280-285, 296-306 **(56.2%)**	103-107, 139-143, 155-159, 301-306 **(6.86%)**	15-23, 30-38, 70-78, 80-90, 101-107, 109-121, 135-141, 143-166, 199-218, 221-225, 227-238, 254-269, 281-287, 295-301 **(51.3%)**	29-44, 56-60, 74-78, 84-89, 102-106, 112-117, 154-165, 200-212, 217-222, 231-235, 260-264 **(27.45%)**	16-24, 66-71, 112-117, 157-162, 199-212, 232-236, 302-306 **(16.6%)**
**NSP6** (290) (YP_009725302.1)	15-31, 48-54, 72-82, 103-108, 113-129, 141-155, 165-	26-30, 35-39, 41-45, 77-84, 90-94, 120-125, 150-154, 165-	10-43, 45-72, 74-86, 88-102, 109-129, 139-156, 161-197,	11-26, 31-45, 47-61, 64-73, 76-86, 88-100, 112-118, 120-127, 138-155,	12-39, 43-59, 66-72, 78-89, 91-95, 115-125, 141-

	226, 241-251, 275-290 **(55.8%)**	169, 171-181, 199-203, 211-219 **(23.79%)**	199-250, 269-281, 283-290 **(82.41%)**	162-170, 180-203, 208-246, 267-271, 273-277 **(67.24%)**	155, 163-172, 176-185, 187-204, 207-232, 237-245 **(57.5%)**
**NSP7** (83) (YP_009725303.1)	6-18, 28-33, 51-63 **(38.55%)**	14-18 **(6%)**	8-19, 29-33, 38-42, 50-63 **(43.37%)**	10-16, 27-31, 37-41, 53-61 **(31.32%)**	9-17, 30-34, 55-61 **(25.3%)**
**NSP8** (198) (YP_009725304.1)	31-36, 44-49, 81-88, 102-107, 114-172, 182-190 **(42.92%)**	129-133, 182-186 **(5%)**	5-9, 11-16, 34-38, 86-96, 122-132, 148-158, 184-191 **(28.78%)**	88-96, 115-122, 127-133, 151-156, 180-188 **(19.69%)**	58-61, 89-93, 128-132, 147-151, 182-187 **(10.6%)**
**NSP9** (113) (YP_009725305.1)	28-33, 61-68, 72-77, 85-90, 101-106 **(28.31%)**	-	38-46, 64-68, 85-94, 100-110 **(30.97%)**	29-33, 40-44, 51-55, 86-92 **(19.46%)**	87-91 **(4.42%)**
**NSP10** (139) (YP_009725306.1)	9-18, 337-47, 50-58, 72-81, 98-103, 107-123 **(45.32%)**	15-19 **(3.59%)**	10-22, 39-48, 54-58, 70-79, 94-99, 109-127 **(45.32%)**	14-21, 42-47, 73-81, 117-122 **(20.86%)**	13-20, 71-78, 116-122 **(15.1%)**
**NSP11** (13) (YP_009725312.1)	8-13 **(46.1%)**	6-10 **(38.46%)**	6-13 **(61.53%)**	-	8-13 **(46.1%)**
**NSP12** (932) (YP_009725308.1)	6-16, 25-32, 34-39, 41-47, 65-73, 75-81, 85-90, 111-	6-10, 34-38, 77-81, 110-114, 146-150, 203-	9-19, 29-37, 39-53, 67-76, 123-132, 142-150, 173-177, 186-	6-10, 30-37, 46-50, 67-73, 100-106, 120-133, 144-151,	201-205, 239-245, 308-323, 335-345,

	116, 142-152, 171-176, 185-193, 198-207, 229-239, 243-249, 313-325, 329-347, 352-357, 359-369, 392-401, 404-411, 421-428, 435-440, 467-480, 490-498, 526-531, 535-540, 554-568, 577-592, 601-609, 632-638, 641-650, 659-664, 670-680, 689-710, 715-720, 762-790, 813-821, 826-831, 842-849, 855-865, 880-888, 925-932	207, 329-333, 337-341, 352-356, 371-375, 491-495, 559-563, 569-573, 585-590, 671-675, 692-697, 775-779, 784-788, 828-832, 843-847, 884-888 **(11.48%)**	192, 199-207, 218-222, 233-242, 244-250, 265-271, 283-287, 310-321, 329-338, 340-351, 366-377, 396-406, 422-430, 436-440, 464-480, 530-535, 538-547, 557-566, 582-594, 596-606, 628-643, 647-652, 662-676, 689-705, 742-792, 838-847, 855-867, 879-888, 899-905, 919-926, 928-932 **(44.52%)**	172-176, 184-188, 190-194, 202-206, 214-218, 238-247, 266-275, 281-292, 306-319, 331-336, 346-350, 370-375, 394-400, 405-409, 438-443, 454-460, 467-474, 492-496, 504-508, 513-517, 526-530, 544-548, 628-639, 650-655, 664-668, 725-733, 743-751, 753-758, 763-768, 784-789, 791-795, 826-830, 842-846, 854-862, 879-890, 900-907, 921-926 **(33.15%)**	395-399, 438-443, 469-474, 559-565, 691-701, 763-768, 784-791, 880-886, 902-906 **(10.4%)**

	**(44.2%)**				
**NSP13** (601) (YP_009725308.1)	1-11, 24-50, 55-65, 67-74, 79-101, 106-111, 120-126, 144-159, 179-185, 187-199, 205-214, 216-233, 235-240, 245-252, 262-267, 289-294, 300-310, 330-362, 366-376, 382-387, 394-401, 421-429, 447-457, 474-486, 491-501, 508-515, 520-526, 528-538, 541-551, 560-578, 587-592, 596-601 **(59%)**	41-45, 191-195, 255-259, 266-270, 295-299, 395-399, 421-425, 478-482, 494-498, 542-546 **(8.31%)**	1-11, 24-34, 36-46, 59-70, 83-95, 107-111, 133-137, 146-156, 177-186, 212-216, 219-233, 243-253, 290-299, 301-313, 319-323, 327-332, 351-363, 367-377, 380-384, 394-401, 420-433,448-454, 471-484, 495-501, 507-513, 522-525, 540-549, 565-578, 585-590 **(46.08%)**	4-8, 18-33, 62-66, 69-73, 120-124, 163-167, 178-187, 223-228, 245-251, 291-300, 303-308, 353-359, 370-374, 395-399, 426-430, 453-457, 470-478, 497-501, 508-513, 542-547, 559-565, 571-577 **(24.45%)**	2-9, 44-48, 65-69, 222-230, 252-256, 292-299, 305-309, 355-361, 543-547, 571-575 **(10.48%)**

**NSP14** (527) (YP_009725309.1)	3-19, 24-33, 35-43, 100-105, 108-127, 159-171, 179-187, 194-202, 205-211, 214-223, 229-236, 240-245, 259-265, 275-280, 285-290, 311-321, 325-333, 346-351, 377-386, 394-402, 416-421, 432-441, 443-448, 455-479, 502-514 **(47%)**	38-42, 114-118, 160-164, 194-201, 232-236, 380-384, 417-421, 459-463, 491-495 **(9.1%)**	9-13, 15-19, 52-65, 82-92, 107-120, 152-157, 159-172, 178-202, 228-238, 240-246, 271-296, 298-303, 312-326, 363-372, 379-389, 400-404, 431-436, 438-442, 444-449, 461-466, 468-479, 495-515 **(45.73%)**	50-60, 71-75, 81-89, 147-155, 159-169, 181-188, 196-200, 207-211, 225-229, 233-243, 245-249, 258-262, 274-282, 284-288, 290-294, 313-317, 348-352, 365-370, 381-387, 397-401, 403-407, 443-447, 491-497, 506-513, 515-522 **(33.20%)**	114-122, 181-187, 224-230, 233-239, 381-387, 442-447, 503-512 **(10.24%)**
**NSP15** (346) (YP_09725310.1)	7-14, 22-43, 51-56, 79-88, 94-106, 114-123, 129-134, 139-156, 162-197, 231-240, 273-280, 285-	23-28, 76-80, 82-86, 100-104, 119-123, 139-144, 164-169, 175-179, 184-188, 275-279, 301-307	1-7, 24-32, 35-39, 51-56, 68-77, 79-85, 96-106, 119-124, 143-147, 163-174, 180-184, 230-241, 245-256, 273-280,	55-59, 83-88, 120-124, 132-136, 211-216, 222- 226, 230-238, 249-253, 277-281, 292-301, 326-333, 339-343 **(21.38%)**	82-86, 98-104, 117-122, 248-252, 292-296, 319-323, 327-332 **(10.98%)**

	298, 301-306, 309-331, 336-342 **(56.93%)**	**(17.34%)**	289-304, 306-334 **(46.24%)**		
**NSP16** (298) (YP_009725311.1)	43-49, 53-58, 66-72, 78-84, 115-122, 124-131, 135-142, 148-158, 162-172, 190-198, 202-210, 225-230, 237-242, 258-263, 270-276, 286-295 **(42.28%)**	27-31, 48-52, 109-113, 124-129, 204-208, 225-229, 278-282 **(12%)**	38-61, 63-71, 84-89, 124-131, 149-161, 163-172, 179-197, 203-211, 222-233, 238-250, 252-261, 269-279, 290-294 **(50%)**	15-23, 48-59, 63-70, 84-90, 124-130, 149-159, 185-195, 204-211, 221-232, 269-274 **(30.53%)**	66-70, 150-156, 186-196, 206-210, 225-231 **(16.1%)**

Supplementary table 4: Predicted APRs in structural proteins of SARS-CoV.

**SARS-CoV Protein (length in amino acids) (UniProt ID)**	**MetAmyl (% of APRs)**	**FISH Amyloid (% of APRs)**	**AGGRESCAN (% of APRs)**	**FoldAmyloid (% of APRs)**	**CamSol (Degree of hydrophobicity)**
**Spike Glycoprotein** (1255) (P59594)	61-76, 87-92, 97-107, 111-130, 146-153, 159-173, 191-207, 211-216, 224-229, 231-239, 249-259, 271-276,	3-10, 39-43, 58-62, 112-118, 160-164, 192-201, 252-256, 292-296, 352-357, 393-397, 403-407, 433-437, 500-	1-12, 54-60, 62-68, 83-88, 97-106, 111-129, 137-142, 152-169, 189-201, 224-239, 247-258, 287-291, 310-316, 318-323, 325-334, 336-341, 349-370, 377-	1-10, 52-64, 99-103, 123-128, 150-155, 183-187, 193-198, 232-237, 251-256, 261-265, 314-318, 353-363, 376-381,	1-10, 59-66, 114-118, 158-162, 194-198, 250-256, 355-364, 418-422, 483-488, 494-501, 525-530, 578-584, 595-599, 657-663, 675-680, 697-706, 734-743,

	287-295, 298-311, 313-323, 328-339, 344-374, 377-394, 399-408, 419-424, 428-433, 456-461, 482-503, 509-514, 523-530, 532-540, 573-586, 594-612, 624-633, 637-646, 659-667, 672-679, 685-690, 700-706, 708-725, 738-745, 763-768, 783-788, 804-812, 837-843, 851-865, 888-900, 912-927, 929-934, 940-945, 951-963, 973-980, 985-995, 7011-1019, 1038-1053, 1057-1063, 1074-1087, 1101-1119, 1141-1146, 1153-1163, 1197-1227, 1230-1237, 1245-1255 **(52.19%)**	504, 516-520, 523-530, 536-540, 545-549, 577-585, 595-599, 765-769, 804-808, 896-900, 935-939, 983-987, 1041-1050, 1075-1079, 1093-1097, 1109-1115, 1157-1162, 1188-1192, 1210-1214 **(14.42%)**	387, 415-425, 471-476, 478-492, 494-506, 508-512, 532-537, 576-585, 592-601, 637-641, 652-665, 669-685, 690-696, 698-709, 711-716, 718-723, 733-741, 743-747, 775-788, 799-815, 835-843, 846-852, 854-878, 880-896, 943-964, 982-995, 1026-1034, 1040-1050, 1073-1084, 1086-1093, 1095-1101, 1106-1119, 1153-1161, 1189-1194, 1196-1231, 1248-1255 **(53.46%)**	417-424, 438-444, 473-478, 493-503, 525-529, 595-599, 661-667, 675-679, 700-704, 720-725, 733-737, 762-766, 780-786, 801-807, 876-889, 975-981, 987-996, 1029-1033, 1042-1049, 1083-1087, 1090-1094, 1109-1114, 1192-1221 **(21.44%)**	854-858, 866-871, 877-881, 986-992, 1041-1050, 1076-1086, 1109-1114, 1193-1224 **(16.09%)**
**Envelope** (76) (P59637)	1-6, 9-63 **(80.26%)**	10-14, 16-23, 25-29 **(23.68%)**	1-5, 11-53, 55-62 **(73.6%)**	11-51, 56-60 **(60.53%)**	12-37, 39-50, 54-59 **(57.89%)**

**Membrane** (221) (P59596)	4-9, 20-27, 34-39, 42-53, 59-72, 74-81, 83-101, 117-132, 135-147, 166-172, 191-196, 215-221 **(55.2%)**	24-28, 43-48, 68-72, 98-102, 107-111, 140-144, 200-204, **(16.28%)**	18-37, 42-57, 59-107, 117-121, 123-130, 134-147, 170-180, 216-221 **(58.37%)**	17-38, 43-59, 61-75, 80-104, 107-111, 117-121, 137-146, 175-179, 196-201, 217-221 **(52.04%)**	20-37, 45-78, 80-99 **(32.57%)**
**Nucleocapsid** (422) (P59595)	13-18, 50-59, 109-114, 129-136, 156-162, 233-240, 242-252, 266-276, 333-338, 347-338, 347-356, 389-397 **(21.8%)**	71-76, 160-164, 239-245, 349-353, 386-390 **(6.63%)**	50-57, 108-117, 127-138, 218-227, 311-323, 328-336, 349-353 **(15.87%)**	52-56, 86-90, 107-114, 131-135, 220-227, 314-318, 330-334, 351-356 **(11.14%)**	52-57, 108-114, 131-135, 331-335 **(5.45%)**

Supplementary table 5: Predicted APRs in accessory proteins of SARS-CoV.

**SARS-CoV Proteins (length in amino acids) (UniProt ID)**	**MetAmyl (% of APRs)**	**FISH Amyloid (% of APRs)**	**AGGRESCAN (% of APRs)**	**FoldAmyloid (% of APRs)**	**CamSol (% of APRs)**
**ORF3a** (274) (P59632)	7-16, 27-36, 45-67, 76-97, 103-119, 143-152, 161-174, 185-190, 196-213, 216-221, 229-237, 242-248, 250-255, 267-274 **(60.58%)**	49-55, 77-81, 86-91, 104-114, 142-146, 214-220, 228-233 **(17.15%)**	1-16, 31-35, 37-41, 43-67, 69-132, 141-150, 160-168, 198-216, 227-236 **(59.48%)**	1-10, 43-57, 61-65, 69-73, 75-97, 103-117, 119-132, 139-152, 154-159, 199-213, 229-235 **(47.08%)**	4-8, 42-57, 76-98, 104-118, 126-131, 143-151, 199-211, 228-234 **(34.30%)**
**ORF3b** (154) (P59633)	3-37, 43-61, 67-76, 79-87, 116-121, 137-148	16-25, 46-50, 116-121 **(13.63%)**	4-34, 44-67, 71-88, 93-97, 116-123 **(55.84%)**	4-8, 12-21, 25-31, 46-52, 61-65, 75-89,	7-30, 46-51, 81-86, 116-122 **(27.92%)**

	**(59.09%)**			112-125, 128-134 **(45.45%)**	
**ORF6** (63) (P59634)	4-26, 28-41 **(58.73%)**	7-11, 14-18, 29-34 **(25.39%)**	1-39 **(61.90%)**	1-10, 12-40 **(61.90%)**	1-19, 24-35 **(49.20%)**
**ORF7a** (122) (P59635)	3-17, 23-32, 56-64, 100-118 **(43.44%)**	5-11, 17-21, 101-105, 108-113 **(18.85%)**	1-18, 26-33, 55-67, 101-118 **(65.9%)**	1-13, 16-22, 63-67, 73-77, 99-117 **(40.16%)**	1-15, 56-66, 100-115 **(34.42%)**
**ORF7b** (44) (Q7TFA1)	5-12, 18-35 **(59%)**	12-28 **(38.63%)**	5-33 **(65.9%)**	5-32 **(63.64%)**	7-32 **(59.09%)**
**ORF8a** (39) (Q7TFA0)	1-25 **(61.53%)**	2-6 **(12.8%)**	1-21 **(53.8%)**	1-22 **(54.41%)**	2-21 **(51.28%)**
**ORF8b** (84) (Q80H93)	2-10, 26-32, 34-49, 73-81 **(48.8%)**	6-10 **(5.95%)**	1-9, 17-28, 33-39, 54-63, 72-80 **(55.95%)**	1-9, 18-23, 28-38, 56-64, 73-79 **(50%)**	17-25, 74-78 **(16.66%)**
**ORF9b** (98) (P59636)	4-9, 19-27, 41-48, 72-81, 90-98 **(42.8%)**	41-46 **(6.12%)**	12-17, 21-25, 45-50, 52-56, 75-80, 91-98 **(36.73%)**	21-26, 43-49 **(13.27%)**	73-79, 93-97 **(12.24%)**
**ORF14** (70) (Q7TLC7)	35-44, 49-54, 58-66 **(35.71%)**	36-44, 48-52 **(20%)**	35-41, 44-48, 50-57, 59-64 **(37.14%)**	35-39, 51-60 **(21.43%)**	33-40 **(11.42%)**

Supplementary table 6: Predicted APRs in non-structural proteins of SARS-CoV.

**SARS-CoV (UniProt ID: P0C6X7) Proteins (length in amino acids)**	**MetAmyl** **(% of APRs)**	**FISH Amyloid** **(% of APRs)**	**AGGRESCAN** **(% of APRs)**	**FoldAmyloid** **(% of APRs)**	**CamSol** **(% of APRs)**
**Nsp1** (180)	2-31, 50-55, 66-74, 82-90, 100-115 **(38.88%)**	4-11, 59-66, 105-109 **(11.66%)**	1-8, 20-31, 52-58, 68-74, 85-93, 98-108, 117-121 **(32.77%)**	2-7, 18-22, 25-29, 68-72, 104-109,116-124 **(20%)**	15-20, 103-109 **(7.22%)**
**Nsp2** (638)	1-6, 29-36, 46-53, 92-108, 118-123, 149-154, 166173, 182-190, 225-239, 279-288, 291-312, 318-332,	38-42, 100-104, 168-172, 292-297, 318-322, 326-330, 362-366, 413-418, 447-52, 458-	97-104, 118-124, 137-141, 182-188, 226-242, 282-286, 291-324, 358-371, 384-393, 400-425, 433-445, 447-461, 464-484, 494-	22-26, 48-52, 58-62, 92-96, 134-139, 150-158, 227-236, 241-245, 271-275, 285-289, 294-299, 365-370, 398-406, 415-420, 431-	227-236, 295-299, 413-424, 468-479, 496-501, 600-608 **(8.46%)**

	339-344, 348-354, 358-372, 384-394, 404-431, 437-442, 447-452, 454-462, 471-488, 494-506, 509-518, 525-533, 536-541, 557-564, 568-574, 576-581, 593-608 **(48.74%)**	462, 470-480, 511-515, 560-564, **(11.59%)**	506, 509-515, 523-534, 572-576, 593-601, 603-612, 625-632 **(38.7%)**	435, 445-452, 466-475, 496-501, 507-511, 527-531, 536-540, 548-552, 606-612, 631-635 **(23.2%)**	
**Nsp3** (1922)	1-10, 12-18, 21-28, 39-67, ,83-90, 103-109, 151-157, 185-191, 197-207, 213-219, 257-263, 273-279, 309-314, 320-337, 342-350, 372-384, 393-405, 444-450, 452-462, 482-488, 508-516, 525-530, 549-561, 569-584, 588-597, 603-616, 628-646, 628-646, 662-667, 681-686, 691-697, 707-712, 724-736, 738-743, 752-757,	5-9, 11-15, 196-205, 319-323, 331-335, 435-439, 455-459, 552-556, 577-581, 588-593, 682-686, 723-727, 762-766, 800-804, 932-927, 941-945, 987-991, 1045-1046, 1083-1087, 1115-1119, 1125-1130, 1149-1153, 1323-1327, 1373-1377, 1410-1414, 1548-1552, 1555-1559, 1592-1596, 1621-1625, 1697-1701, 1787-1791, 1799-1803,	43-47, 52-67, 83-91, 102-108, 144-148, 193-205, 215-222, 258-267, 273-279, 302-306, 308-317, 319-334, 345-351, 393-397, 408-418, 444-459, 480-485, 507-514, 526-534, 549-562, 571-587, 589-601, 608-613, 631-637, 643-647, 660-665, 695-699, 713-717, 719-734, 742-748, 774-780, 819-825, 833-846, 868-879, 905-912, 922-934, 937-944, 958-968, 1022-1032, 1039-1046, 1113-1119, 1129-1134, 1149-1154, 1165-1174, 1229-1235,	23-27, 53-58, 84-92, 101-107, 200-205, 215-219, 289-293, 303-307, 323-328, 331-336, 343-350, 411-416, 443-447, 455-459, 510-514, 529-538, 547-559, 578-584, 591-595, 608-613, 622-626, 670-674, 683-687, 692-696, 728-732, 776-780, 791-796, 802-807, 833-843, 856-861, 868-876, 892-896, 907-912, 926-931, 1024-1028, 1116-1120, 1127-1133, 1149-1153, 1166-1174, 1232-1236, 1321-1326, 1328-1341, 1384-1407, 1409-	54-60, 85-89, 215-221, 322-328, 331-336, 510-514, 527-531, 577-583, 605-614, 744-748, 835-842, 869-875, 908-912, 943-947, 1150-1154, 1168-1172, 1326-1341, 1387-1414, 1447-1451, 1465-1472, 1476-1481, 1485-1503, 1505-1516, 1519-1531, 1541-1560, 1566-1570, 1697-1702, 1718-1723, 1831-1835 **(12.90%)**

	760-766, 775-782, 8181-826, 833-840, 844-850, 868-885, 905-914, 918-927, 938-949, 952-967, 994-1001, 1018-1037, 1044-1051, 1102-1109, 1113-1119, 1124-1131, 1148-1162, 1182-1194, 1220-1237, 1240-1247, 1261-1266, 1279-1291, 1296-1302, 1306-1315, 1319-1345, 1359-1367, 1372-1378, 1383-1389, 1396-1416, 1420-1425, 1432-1438, 1444-1453, 1463-1473, 1475-1482, 1487-1492, 1501-1512, 1514-1519, 1525-1533, 1540-1560, 1562-1572, 1580-1590, 1592-1600, 1614-1625, 1645-1658, 1676-1683, 1695-1705, 1705-1723, 1735-1750, 1752-1765, 1769-1775, 1787-1814,	1897-1871, 1909-1914, **(9.26%)**	1258-1263, 1273-1292, 1294-1302, 1304-1310, 1323-1328, 1330-1341, 1360-1375, 1381-1418, 1440-1445, 1447-1456, 1462-1534, 1536-1562, 1564-1570, 1581-1585, 1587-1600, 1616-1624, 1648-1655, 1697-1704, 1715-1726, 1728-1735, 1747-1763, 1765-1776, 1783-1797, 1833-1838, 1866-1877, 1904-1922 **(41.4%)**	1414, 1447-1452, 1477-1482, 1484-1501, 1504-1532, 1538-1559, 1568-1572, 1591-1596, 1609-1613, 1647-1651, 1660-1665, 1678-1682, 1697-1702, 1718-1733, 1790-1794, 1811-1816, 1831-1836, 1868-1873, 1897-1903 **(23.99%)**	

	1823-1829, 1834-1839, 1857-1862, 1865-1871, 1897-1902, 1905-1917 **(49.58%)**				
**Nsp4** (500)	1-8, 13-36, 53-58, 86-104, 109-114, 120-137, 147-152, 154-161, 166-171, 208-218, 229-239, 250-278, 280-302, 307-312, 323-367, 372-387, 393-398, 402-412, 444-449, 483-488, 491-500 **(56.2%)**	22-26, 52-56, 90-95, 117-121, 128-133, 229-233, 257-261, 264-268, 325-329, 336-347, 349-353, 358-362, 377-385, 406-410, 419-423, 444-448, 484-491 **(20.2%)**	1-28, 30-35, 90-103, 113-122, 124-135, 137-159, 170-181, 193-201, 203-217, 230-240, 250-269, 275-307, 309-313, 315-332, 334-338, 340-370, 375-399, 401-427, 430-436, 439-453, 494-500 **(72.4%)**	7-35, 72-76, 92-97, 99-104, 118-127, 190-194, 211-215, 253-257, 262-270, 290-307, 319-333, 342-354, 358-371, 373-387, 389-405, 417-422, 426-430, 436-440, 442-449 **(39.2%)**	3-8, 14-33, 90-94, 119-124, 128-132, 253-258, 264-272, 286-302, 316-335, 337-354, 360-387, 390-395, 417-421, 495-500 **(31.4%)**
**Nsp5** (306)	13-26, 35-47, 63-82, 89-94, 101-118, 123-128, 141-162, 166-176, 196-215, 223-228, 246-251, 254-262, 280-287, 296-306 **(55.55%)**	65-69, 103-107, 139-143, 155-159, 301-306 **(8.49%)**	15-23, 31-35, 68-78, 80-84, 101-107, 109-121, 143-166, 199-218, 221-225, 227-238, 254-269, 281-287, 295-301 **(46.07%)**	29-33, 36-44, 56-60, 74-78, 84-89, 102-106, 112-117, 154-165, 200-212, 217-222, 231-235, 260-264 **(26.8%)**	16-24, 65-71, 112-117, 157-162, 201-212, 232-236, 302-306 **(16.33%)**
**Nsp6** (290)	6-11, 19-40, 45-52, 72-82, 113-118, 123-129, 141-155, 164-185, 187-197, 199-226, 241-251, 264-269, 283-290	17-30, 33-37, 41-45, 77-84, 90-94, 120-125, 150-154, 171-183, 211-219, **(24.13%)**	9-43, 45-72, 74-86, 88-102, 110-129, 139-156, 161-197, 199-250, 267-281, 283-290 **(83.44%)**	11-26, 31-38, 40-45, 47-60, 64-73, 76-86, 88-99, 112-127, 138-155, 162-170, 180-203, 208-246, 267-271, 273-277 **(66.56%)**	12-29, 31-40, 43-58, 66-73, 77-89, 91-95, 115-125, 141-153, 163-172, 176-204, 207-232, 237-245 **(57.93%)**

	**(55.51%)**				
**Nsp7** (83)	6-18, 28-33, 51-63 **(38.55%)**	14-18 **(6%)**	8-19, 29-33, 38-42, 50-63 **(43.37%)**	10-16, 27-31, 37-41, 53-61 **(31.33%)**	9-17, 30-34, 55-61 **(25.30%)**
**Nsp8** (198)	31-36, 44-49, 81-88, 102-107, 114-124, 126-135, 145-172, 182-190 **(42.42%)**	182-186 **(3.62%)**	5-9, 11-15, 34-38, 86-96, 122-132, 148-158, 184-191 **(28.2%)**	88-96, 115-122, 127-131, 151-156, 180-188 **(18.69%)**	89-93, 128-133, 147-151, 182-187 **(11.11%)**
**Nsp9** (113)	28-33, 61-68, 72-77, 85-90, 101-106 **(28.31%)**	-	39-45, 64-68, 85-94, 100-110 **(30.08%)**	29-33, 40-44, 51-55, 86-92 **(19.47%)**	87-91 **(4.42%)**
**Nsp10** (139)	9-18, 37-47, 50-58, 72-81, 98-103, 107-123 **(45.32%)**	15-19 **(3.59%)**	10-22, 39-48, 54-58, 70-79, 94-99, 113-127 **(42.44%)**	14-20, 42-47, 73-81, 117-122 **(20.14%)**	13-19, 73-78, 116-122 **(14.38%)**
**NSP11 (13)**	4-13 **(76.9%)**	6-10 **(38.46%)**	6-13 **(61.53%)**	6-10 **(38.46%)**	8-13 **(46.1%)**
**Nsp12** (932)	4-16, 25-32, 34-39, 41-47, 66-73, ,85-91, 93-98, 111-116, 142-152, 171-176, 185-193, 198-207, 220-239, 243-49, 313-325, 329-347, 352-357, 359-369, 392-401, 404-411, 421-428, 435-440, 467-480, 490-498, 526-531, 535-540, 554-568, 577-592, 601-609, 632-638, 659-664, 670-680,	34-38, 77-81, 110-114, 146-150, 203-207, 230-234, 329-333, 337-341, 352-356, 371-375, 491-495, 559-563, 569-573, 585-590, 671-675, 692-697, 775-779, 784-788, 828-832, 843-847, 884-888 **(11.48%)**	7-19, 29-37, 39-53, 66-75, 89-93, 97-103, 123-132, 142-150, 173-177, 187-192, 199-207, 218-226, 233-242, 244-250, 265-271, 282-287, 310-321, 329-338, 340-351, 366-377, 396-406, 422-430, 436-440, 464-480, 530-535, 538-547, 557-566, 582-594, 596-607, 628-638, 662-676, 689-705, 742-763, 773-792, 838-847, 855-867, 879-888, 899-905, 919-926, 928-932 **(44.7%)**	6-10, 30-37, 46-50, 67-73, 99-105, 120-133, 144-151, 172-176, 190-194, 202-206, 214-218, 238-247, 266-275, 280-292, 306-319, 331-336, 346-350, 370-375, 394-400, 405-409, 438-443, 454-460, 467-474, 492-496, 504-508, 513-517, 526-530, 544-548, 628-639, 650-655, 664-668, 725-733, 743-751, 753-758, 783-789, 791-795, 826-830, 842-846, 854-862, 879-890, 900-907, 921-926	239-245, 308-320, 335-345, 395-399, 438-443, 469-474, 560-565, 691-701, 785-791, 880-886, 902-906 **(9.01%)**

	689-710, 715-720, 742-747, 761-769, 773-780, 782-790, 813-821, 842-849, 855-865, 880-888, 925-932 **(44.52%)**			**(32.19%)**	
**Nsp13** (601)	1-11, 24-50, 55-65, 67-74, 79-101, 106-111, 120-126, 144-159, 179-185, 187-199, 205-214, 216-233, 235-240, 245-252, 262-267, 289-294, 300-310, 330-336, 346-376, 382-387, 394-401, 421-429, 447-457, 474-486, 491-501, 508-515, 520-526, 528-538, 541-551, 560-568, 570-578, 587-592, 596-601 **(58.9%)**	41-45, 191-195, 255-259, 266-270, 295-299, 395-399, 421-425, 478-482, 494-498, 542-546 **(8.31%)**	1-11, 24-34, 36-46, 59-70, 83-95, 107-111, 133-137, 146-156, 177-186, 212-216, 219-233, 243-253, 290-299, 301-313, 319-323, 327-332, 351-363, 367-377, 380-384, 394-401, 420-433, 448-454, 471-484, 495-501, 507-513, 522-528, 540-549, 565-578, 585-590 **(46.08%)**	4-8, 18-33, 62-66, 69-73, 120-124, 163-167, 178-187, 223-228, 245-251, 291-300, 303-308, 353-359, 370-374, 395-399, 426-430, 453-457, 470-478, 497-501, 508-513, 542-547, 559-565, 571-577 **(24.46%)**	2-9, 44-48, 65-69, 222-230, 252-256, 292-299, 305-309, 354-361, 543-547, 571-575 **(10.48%)**
**Nsp14** (527)	3-19, 24-43, 100-105, 108-128, 159-171, 179-187, 194-202, 205-211, 214-224,	38-42, 114-118, 160-164, 194-201, 232-236, 293-297, 380-384, 417-421, 459-	9-19, 28-38, 52-65, 82-92, 107-120, 152-157, 159-172, 178-202, 228-238, 240-246, 271-296, 312-326, 363-369,	50-60, 71-75, 81-89, 147-155, 159-169, 181-188, 196-200, 225-229, 233-243, 245-249, 274-282, 284-288, 313-	28-32, 114-122, 181-187, 226-230, 233-239, 380-387, 442-447, 503-512 **(10.81%)**

	228-236, 240-245, 275-280, 285-290, 307-322, 325-333, 346-351, 377-386, 395-402, 416-421, 434-439, 443-448, 455-479, 502-512 **(56.9%)**	463, 507-511 **(10.05%)**	379-389, 396-404, 444-449, 461-466, 468-479, 496-515 **(44.78%)**	317, 348-352, 365-370, 381-387, 396-401, 403-407, 443-447, 472-477, 492-497, 506-513, 515-522 **(30.36%)**	
**Nsp15** (346)	5-14, 22-43, 51-56, 79-88, 94-106, 116-125, 129-134, 139-153, 160-187, 190-197, 231-240, 273-280, 285-298, 301-306, 309-331, 336-342 **(56.64%)**	23-28, 34-43, 65-69, 76-80, 82-86, 95-104, 119-123, 139-144, 164-169, 175-179, 181-186, 275-279, 301-307 **(23.41%)**	1-7, 24-33, 35-40, 51-56, 68-77, 79-85, 96-106, 116-124, 143-148, 162-177, 180-184, 216-222, 230-237, 245-254, 273-280, 289-304, 306-334 **(49.44%)**	55-59, 83-88, 120-124, 132-136, 212-216, 222-226, 230-238, 249-253, 277-281, 292-301, 326-333, 339-343 **(21.09%)**	82-86, 97-102, 118-122, 248-252, 291-296, 319-323 **(9.53%)**
**Nsp16** (298)	34-39, 43-49, 53-58, 66-72, 78-84, 115-122, 124-131, 148-157, 162-172, 190-198, 2020-208, 225-230, 237-242, 258-263, 270-275, 285-297 **(41.27%)**	27-31, 48-52, 109-113, 124-129, 204-208, 225-229, 278-282, **(9.04%)**	38-61, 63-71, 84-89, 124-131, 149-161, 163-172, 179-197, 203-211, 225-233, 238-250, 252-260, 270-279, 290-294 **(48.32%)**	15-23, 48-59, 63-70, 84-90, 124-130, 149-156, 185-195, 226-232, 269-274 **(25.17%)**	50-62, 66-70, 150-156, 186-196, 225-231 **(14.42%)**

Supplementary table 7: NetChop 3.1 prediction of 20S proteasome cleavage site in structural proteins of SARS-CoV-2. The red colored residues represent the cleavage site while the underlined and bold residues are APRs predicted by FISH Amyloid server.

Protein Name	Distribution of predicted 20s proteasome cleavage sites	Distribution of cleavage sites in aggregation prone regions (APR)
**Spike**	1-MFVFLVLLPLVSSQCVNLTTRTQLPPAYTN-30<br>31-SFTRGVYYPDKVFRSSVLHSTQDLFLPFFS-60<br>61-NVTWFHAIHVSGTNGTKRFDNPVLPFNDGV-90<br>91-YFASTEKSNIIRGWIFGTTLDSKTQSLLIV-120<br>121-NNATNVVIKVCEFQFCNDPFLGVYYHKNNK-150<br>151-SWMESEFRVYSSANNCTFEYVSQPFLMDLE-180<br>181-GKQGNFKNLREFVFKNIDGYFKIYSKHTPI-210<br>211-NLVRDLPQGFSALEPLVDLPIGINITRFQT-240<br>241-LLALHRSYLTPGDSSSGWTAGAAAYYVGYL-270<br>271-QPRTFLLKYNENGTITDAVDCALDPLSETK-300<br>301-CTLKSFTVEKGIYQTSNFRVQPTESIVRFP-330<br>331-NITNLCPFGEVFNATRFASVYAWNRKRISN-360<br>361-CVADYSVLYNSASFSTFKCYGVSPTKLNDL-390<br>391-CFTNVYADSFVIRGDEVRQIAPGQTGKIAD-420<br>421-YNYKLPDDFTGCVIAWNSNNLDSKVGGNYN-450<br>451-YLYRLFRKSNLKPFERDISTEIYQAGSTPC-480<br>481-NGVEGFNCYFPLQSYGFQPTNGVGYQPYRV-510<br>511-VVLSFELLHAPATVCGPKKSTNLVKNKCVN-540<br>541-FNFNGLTGTGVLTESNKKFLPFQQFGRDIA-570<br>571-DTTDAVRDPQTLEILDITPCSFGGVSVITP-600<br>601-GTNTSNQVAVLYQDVNCTEVPVAIHADQLT-630<br>631-PTWRVYSTGSNVFQTRAGCLIGAEHVNNSY-660<br>661-ECDIPIGAGICASYQTQTNSPRRARSVASQ-690<br>691-SIIAYTMSLGAENSVAYSNNSIAIPTNFTI-720<br>721-SVTTEILPVSMTKTSVDCTMYICGDSTECS-750<br>751-NLLLQYGSFCTQLNRALTGIAVEQDKNTQE-780<br>781-VFAQVKQIYKTPPIKDFGGFNFSQILPDPS-810<br>811-KPSKRSFIEDLLFNKVTLADAGFIKQYGDC-840<br>841-LGDIAARDLICAQKFNGLTVLPPLLTDEMI-870<br>871-AQYTSALLAGTITSGWTFGAGAALQIPFAM-900<br>901-QMAYRFNGIGVTQNVLYENQKLIANQFNSA-930<br>931-IGKIQDSLSSTASALGKLQDVVNQNAQALN-960<br>961-TLVKQLSSNFGAISSVLNDILSRLDKVEAE-990<br>991-VQIDRLITGRLQSLQTYVTQQLIRAAEIRAS-1020<br>1021-ANLAATKMSECVLGQSKRVDFCGKGYHLMS-<br>1050<br>1051-FPQSAPHGVVFLHVTYVPAQEKNFTTAPAI-1080<br>1081-CHDGKAHFPREGVFVSNGTHWFVTQRNFYE-<br>1110<br>1111-PQIITTDNTFVSGNCDVVIGIVNNTVYDPL-1140<br>1141-QPELDSFKEELDKYFKNHTSPDVDLGDISG-1170<br>1171-INASVVNIQKEIDRLNEVAKNLNESLIDLQ-1200<br>1201-ELGKYEQYIKWPWYIWLGFIAGLIAIVMVT-1230<br>1231-IMLCCMTSCCSLKGCCSCGSCCKFDEDDS-1260<br>1261-EPVLKGVKLHYT-1273	Total sites: 196 (15%)<br>Sites within APR: 34 (17.34%)<br>Sites outside APR: 162 (82.65%)
**Envelope**	1-MYSFVSEETGTLIVNSVLLFLAFVVFLLVT-30<br>31-LAILTALRLCAYCCNIVNVSLVKPSFYVYS-60	Total sites: 17 (22%)

	61-RVKNLNSSRVPDLLV-75	Sites within APR: 6 (35.30%)<br>Sites outside APR: 11 (64.70%)
**Membrane**	1-MADSNGTITVEELKKLLEQWNLVI**GFLFL**T-30<br>31-WICLLQFAYANRN**RFLYIIKLIFLWL**LWPV-60<br>61-TLACFVLA**AVYRI**NWITGGIAIAMACLVGL-90<br>91-MWLSYFIA**SFRLF**ARTR**SMWSF**NPETNILL-120<br>121-**NVPL**HGTILTRPLLESELVIGAVILRGHLR-150<br>151-IAGHHLGRCDIKDLPKEITVATSRTLSYYK-180<br>181-LGASQRVAGDSGFAAYSRYRIGNYKLNTDH-210<br>211-SSSSDNIALLVQ-222	Total sites: 58 (26%)<br>Sites within APR: 16 (27.59%)<br>Sites outside APR: 42 (72.41%)
**Nucleocapsid**	1-MSDNGPQNQRNAPRITFGGPSDSTGSNQNG-30<br>31-ERSGARSKQRRPQGLPNNTASWFTALTQHG-60<br>61-KEDLKFPRG**QGVPIN**TNSSPDDQIGYYRRA-90<br>91-TRRIRGGDGKMKDLSPRWYFYYLGTGPEAG-120<br>121-LPYGANKDGIIWVATEGALNTPKDHIGTRN-150<br>151-PANNAAIV**LQLPQ**GTTLPKGFYAEGSRGGS-180<br>181-QASSRSSSRSRNSSRNSTPGSSRGTSPARM-210<br>211-AGNGGDAALALLLLDRLNQLESKMSGK**GQQ**-240<br>241-**QQGQ**TVTKKSAAEASKKPRQKRTATKAYNV-270<br>271-TQAFGRRGPEQTQGNFGDQELIRQGTDYKH-300<br>301-WPQIAQFAPSASAFFGMSRIGMEVTPSGTW-330<br>331-LTYTGAIKLDDKDPNFK**DQVIL**LNKHIDAY-360<br>361-KTFPPTEPKKDKKKKADETQALPQ**RQKKQ**Q-390<br>391-TVTLLPAADLDDFS**KQLQQ**SMSSADSTQA-419	Total sites: 39 (9.30%)<br>Sites within APR: 4 (10.25%)<br>Sites outside APR: 35 (89.74%)

Supplementary table 8: NetChop 3.1 prediction of 20S proteasome cleavage site in accessory proteins of SARS-CoV-2. The red colored residues represent the cleavage site while the underlined and bold residues are APRs predicted by FISH Amyloid server.

Protein Name	Distribution of predicted 20s proteasome cleavage sites	Distribution of cleavage sites in aggregation prone regions (APR)
**ORF3A**	1-MDLFMRIFTIGTVTLKQGEIKDATPSDFVR-30<br>31-ATATIPIQASLPF**GWLIVGVALL**AVFQSAS-60<br>61-KIITLKKRWQLALSK**GVHFV**CNLLL**LFVTV**-90<br>91-YSHLLLVAAGLEAPFLYL**YALVYFLQS**I**NF**-120<br>121-**VRIIM**RLWLCWKCRSKNPLLY**DANYF**LCWH-150<br>151-TNCYDYCIPYNSVTS**SIVIT**SGDGTTSPIS-180<br>181-EHDYQIGGYTEKWESGVKDCVVLHSYFTSD-210<br>211-Y**YQLYS**TQLSTDT**GVEHVTFFIY**NKIVDEP-240<br>241-EEHVQIHTIDGSS**GVVNP**VMEPIYDEPTTT-270<br>271-TSVPL-275	Total sites: 50 (18.18%)<br>Sites within APR: 17 (34.0%)<br>Sites outside APR: 33 (66.0%)
**ORF3B**	1-MMPTIFFA**GILIVTTIV**YLTIV-22	Total sites: 6 (27.2%)

		Sites within APR: 2 (33.33%)<br>Sites outside APR: 4 (66.66%)
**ORF6**	1-MFHLVD**FQVTI**AE**ILLIIM**RTFKVSIW**NLD**-30<br>31-**YIINLII**KNLSKSLTENKYSQLDEEQPMEI-60<br>61-D	Total sites: 10 (16.39%)<br>Sites within APR: 6 (60.0%)<br>Sites outside APR: 4 (40%)
**ORF7A**	1-MKII**LFLAL**ITLATCE**LYHYQ**ECVRGTTVL-30<br>31-LKEPCSSGTYEGNSPFHPLADNKFALTCFS-60<br>61-TQFAFACPDGVKHVYQLRARSVSPKLFIRQ-90<br>91-EEVQELYSP**IFLIVAAIVFIT**LCFTLKRKT-120<br>121-E	Total sites: 20 (16.52%)<br>Sites within APR: 9 (45.0%)<br>Sites outside APR: 11 (55.0%)
**ORF7B**	1-MIELSLIDFYL**CFLAFLLFLVLIMLIIF**WF-30<br>31-SLELQDHNETCHA-43	Total sites: 19 (44.18%)<br>Sites within APR: 12 (63.16%)<br>Sites outside APR: 7 (36.84%)
**ORF8**	1-M**KFLVFLGII**TTVAAFHQECSLQSCTQHQP-30<br>31-YVVDDPCPIHFYSKWYIRVGARKSAPLIEL-60<br>61-CVDEAGSKSPIQYIDI**GNYTV**SCLPFTINC-90<br>91-QEPKLGSLVVRCSFYE**DFLEY**HDVRVVLDF-120<br>121-I	Total sites: 19 (15.70%)<br>Sites within APR: 5 (26.31%)<br>Sites outside APR:14 (73.68%)
**ORF9B**	1-MDPKISEMHPALRLVDPQIQLAVTRMENAV-30<br>31-GRDQNNVGP**KVYPII**LRLGSPLSLNMARKT-60<br>61-LNSLEDKAFQLTPIAVQMTKLATTEELPDE-90<br>91-FVVVTVK-97	Total sites: 12 (12.37%)<br>Sites within APR: 1 (8.33%)<br>Sites outside APR:11 (91.66%)
**ORF10**	1-MGYINVFAFPFTIYSLLLCRMN**SRNYI**AQV-30<br>31-**DVVN**FNLT-38	Total sites: 9 (23.68%)<br>Sites within APR: 2 (22.22%)<br>Sites outside APR:7 (77.77%)
**ORF14**	1-MLQSCYNFLKEQHCQKASTQKGAEAAVKPL-30<br>31-LVPHHVVA**TVQEIQ**LQAAVGELLLLEWLAM-60<br>61-AVMLLLLCCCLTD-73	Total sites: 13 (17.80%)<br>Sites within APR: 1 (7.69%)<br>Sites outside APR: 12 (92.31%)

Supplementary table 9: NetChop 3.1 prediction of 20S proteasome cleavage site in non-structural proteins of SARS-CoV-2. The red colored residues represent the cleavage site while the underlined and bold residues are APRs predicted by FISH Amyloid server.

Protein Name	Distribution of predicted 20s proteasome cleavage sites	Distribution of cleavage sites in aggregation prone regions (APR)
**NSP1**	1-MESLVPGFNEKTHVQLSLPVLQVRDVLVRG-30<br>31-FGDSVEEVLSEARQHLKDGTCGLVEVEK**GV**-60	Total sites: 19 (10.5%)

	61-**LPQLEQ**PYVFIKRSDARTAPHGHVMVELVA-90<br>91-E**LEGIQ**YGRSGET**LGVLVPHVGEI**PVAYRK-120<br>121-VLLRKNGNKGAGGHSYGADLKSFDLGDELG-150<br>151-TDPYEDFQENWNTKHSSGVTRELMRELNGG-180	Sites within APR: 1 (5.26%)<br>Sites outside APR: 18 (94.74%)
**NSP2**	1-AYTRYVDNNFCGPDGYPLECIKDLLARAGK-30<br>31-ASCTLSEQLDFIDTKRGVYCCREHEHEIAW-60<br>61-YTERSEKSYELQTPFEIKLAKKFDTFNGEC-90<br>91-PNFVFPLNSIIKTIQPRVEKKKLDGFMGRI-120<br>121-RSVYPVASPNECNQMCLSTLMKCDHCGETS-150<br>151-WQTGDFVKATCEFCGTENLTKEGATTCGYL-180<br>181-PQN**AVVKIY**CPACHNSEVGPEHSLAEYHNE-210<br>211-SGLKTILRKGGRTIAFGGCVFSYVGCHNKC-240<br>241-AYWVPRASANIGCNHT**GVVGE**GSEGLNDNL-270<br>271-LEILQKEKVNINIVGDFKLNEEIAIILASF-300<br>301-SASTSAFVETVKGLDYKAFKQIVESCGNFK-330<br>331-VTKGKAKKGAWNIGEQKSILSPLYAFASEA-360<br>361-AR**VVRSIF**SRTLETAQNSVRVLQKAAITIL-390<br>391-DGISQYSLRLIDAMMFTSDLATNNLVVMAY-420<br>421-ITG**GVVQL**TSQW**LTNIFGTV**YEKLKPVLDW-450<br>451-LEEKFKE**GVEFL**RDGWE**IVKFI**STCACEIV-480<br>481-GGQIVTCAKEIKE**SVQTF**FKLVNKFLALCA-510<br>511-DSIIIGGAKLKALNLGETFVTHSKGLYRKC-540<br>541-VKSREETGLLMPLKAPKEI**IFLEG**ETLPTE-570<br>571-VLTEEVVLKTGDLQPLEQPTSEAVEAPLVG-600<br>601-TPVCINGLMLLEIKDTEKYCALAPNMMVTN-630<br>631-NTFTLKGG-638	Total sites: 76 (11.91%)<br>Sites within APR: 7 (9.21%)<br>Sites outside APR:69 (90.79%)
**NSP3**	1-APTKVTFGD**DTVIE**VQGYK**SVNIT**FELDER-30<br>31-IDKVLNEKCSAYTVELGTEVNEFACVVADA-60<br>61-VIKTLQPVSELLTPLGIDLDEWSMATYYLF-90<br>91-DESGEFKLASHMYCSFYPPDEDEEEGDCEE-120<br>121-EEFEPSTQYEYGTEDDYQGKPLEFGATSAA-150<br>151-LQPEEEQEEDWLDDDSQQTVGQQDGSEDNQ-180<br>181-TTTIQTIVEVQPQLEMELTP**VVQTI**EVN**SF**-210<br>211-**SGY**LKLT**DNVYI**KNADIVEEAKKVKPTVVV-240<br>241-NAANVYLKHGGGVAGALNKATNNAMQVESD-270<br>271-DYIATNGPLKVGGSCVLSGHNLAKHCLHVV-300<br>301-GPNVNKGEDIQLLKSAYENFNQHEVLLAPL-330<br>331-LSAGIFGADPIHSLRVCVDTVRTNVYLAVF-360<br>361-DKNLYDKLVS**SFLEM**KSE**KQVEQ**KIAEIPK-390<br>391-EEVKPFITESKPSVEQRKQDDKKIKACVEE-420<br>421-VTTTLEETKFLTE**NLLLYI**DINGNLHPDSA-450<br>451-T**LVSDI**DITFLKKDAPYIVGDVVQE**GVLTA**-480<br>481-VVIPTKKAGGTTEMLAKALRKVP**TDNYI**TT-510<br>511-YPGQGLNGYTVEEAKTVLKKCK**SAFYI**LPS-540<br>541-IISNEKQEILGTVSWNLREMLAHAEETRKL-570<br>571-MPVCVETKA**IVSTI**QRKYKGIKIQE**GVVDY**-600<br>601-GARFYFYTSKTTVASLINTLNDLNETLVTM-630	Total sites: 293 (15.05%)<br>Sites within APR: 51 (17.40%)<br>Sites outside APR: 242 (82.60%)

	631-PLGYVTHGLNLEEAARYMRSLKVPATVSVS-660<br>661-SPDAVTAYNGYLTSSSKTPEEHFIETISLA-690<br>691-GSYKDWSYSGQSTQLGIEFLKRGDK**SVYYT**-720<br>721-SNPTTFHLDGEVITFDNLKTLLSLR**EVRTI**-750<br>751-KVFT**TVDNI**NLHTQVVDMSMTYGQQFGPTY-780<br>781-LD**GADVT**KIKPHNSHEGKTFYVLPNDDTLR-810<br>811-VEAFEYYHTTDPSFLGRYMSALNHTKKWKY-840<br>841-PQVNGLTSIKWADNNCYLATALLTLQQIEL-870<br>871-KFNPPALQDAYYRARAGEAANFCALILAYC-900<br>901-NKTVGELGDVRETMSYLFQHANLDSCKRVL-930<br>931-NVVCKTCGQQQTTLK**GVEAV**MYMGTLSYEQ-<br>960<br>961-FKK**GVQIP**CTCGKQATKYLVQQESPFVMMS-990<br>991-APPAQYELKHGTFTCASEYT**GNYQC**GHYKH-<br>1020<br>1021-ITSKETLYCIDGALLTKSSEYKGPITDVFY-1050<br>1051-KENSYTTTIKPVTYKLD**GVVCT**EIDPKLDN-<br>1080<br>1081-YYKKDNSYFTEQPIDLVPNQPYPNA**SFDNF**-<br>1110<br>1111-KFVCDNIKFADDLNQLTGYKKPASRELKVT-<br>1140<br>1141-FFPDLNG**DVVAID**YKHYTPSFKKGAKLLHK-<br>1170<br>1171-PIVWHVNNATNKATYKPNTWCIRCLWSTKP-<br>1200<br>1201-VETSNSFDVLKSEDAQGMDNLACEDLKPVS-<br>1230<br>1231-EEVVENPTIQKDVLECNVKTT**EVVGDI**IL-1260<br>1261-KPANNSLKITEEVGHTDLMAAYVDNSSLTI-<br>1290<br>1291-KKPNELSRVLGLKTLATHGLAAVNSVPWDT-<br>1320<br>1321-IANYAKPFLNKVVS**TTTNI**VTRCLNRVCTN-<br>1350<br>1351-YMPYFFTLLLQLCTFTRSTNSRIKASMPTT-1380<br>1381-IAKNTVKSVGKFCLE**ASFNY**LKSPNFSK**LI**-1410<br>1411-**NII**IWFLLLSVC**LGSLIY**STAA**LGVLM**SNL-1440<br>1441-GMPSYCTGYREGYLN**STNVT**IATYCTGSIP-1470<br>1471-CSVCLSGLDSLDTYPSLETIQITISSFKWD1500<br>1501-LTAFGLVAE**WFLAY**ILFT**RFFYV**LGLAAIM-<br>1530<br>1531-QLFFSY**FAVHF**ISNSW**LMWLI**INLVQMAPI-<br>1560<br>1561-SAMVRMYIFF**ASFYYVW**KSYVHVVDGCNSS-<br>1590<br>1591-TCMMCYKRNRATRVECTTIVNGVR**RSFYYV**Y-<br>1620<br>1621-ANGGKGFCKLHNWNCVNCDTFCA**GSTFI**SD-<br>1650<br>1651-EVARDLSLQFKRPINPTDQSSYIVDSVTVK-1680	

	1681-NGSIHLYFDKAGQKTYERHSLSHFVNLDNL-1710<br>1711-RANNTKGSL**PINVI**VFDGKSKCEESSAKSA-1740<br>1741-SVYYSQLMCQPILLLDQALVSDVGDSAEVA-1770<br>1771-VKMFD**AYVNT**FSSTFNVPMEKLKTLVATAE-1800<br>1801-AELAKNVSLDNV**LSTFI**SAARQGFVDSDVE-1830<br>1831-TKDVVECLKLSHQSDIEVTGDSCN**NYMLT**Y-1860<br>1861-NKVENMTPRDLGACIDCSARHINAQVAKSH-1890<br>1891-NIALIWNVKDFMSLSEQLRKQIRSAAKKNN-1920<br>1921-LPFKLTCATTRQVVNVVTTKIALKGG-1946	
**NSP4**	1-KIVNNWLKQLIKVTL**VFLFVAAIF**YLITPV-30<br>31-HVMSKHTDF**SSEII**GYKAIDG**GVTRD**IAST-60<br>61-DTCFANKHADFDTWFSQRGGSYTNDKACPL-90<br>91-IAAVITREVGFVVPGLPGTILRTTNG**DFLH**-120<br>121-**F**LPRVFS**AVGNIC**YTPSKLIEYTDFATSAC-150<br>151-VLAAECTIFKDASGKPVPYCYDTNVLEGSV-180<br>181-AYESLRPDTRYVLMDGSIIQFPNTYLEGSV-210<br>211-RVVTTFDSEYCRHGTCERSEAGVCVSTSGR-240<br>241-WVLNNDYYRSLPGVFC**GVDAV**NLLTNMFTP-270<br>271-LIQPIGALDISASIVAG**GIVAIV**VTC**LAYY**-300<br>301-**F**MRFRRAFGEYSHVVAFNTLLFLMSFTVLC-330<br>331-LTPVY**SFLPGVYSVIYL**YLTFYLTNDV**SFL**-360<br>361-**AH**IQWMVMFTPLVPFWITIAYIICISTKHF-390<br>391-YWFFSNYLKRRV**VFNGVSFSTF**EEAALC**TF**-420<br>421-**LLN**KEMYLKLRSDVLLPLTQYNR**YLALY**NK-450<br>451-YKYFSGAMDTTSYREAACCHLAKALNDFSN-480<br>481-SGS**DVLYQPPQ**TSITSAVLQ-500	Total sites: 109 (21.8%)<br>Sites within APR: 20 (18.34%)<br>Sites outside APR: 89 (81.66%)
**NSP5**	1-SGFRKMAFPSGKVEGCMVQVTCGTTTLNGL-30<br>31-WLDDVVYCPRHVICTSEDMLNPNYEDLLIR-60<br>61-KSNHNFLVQAGNVQLRVIGHSMQNCVLKLK-90<br>91-VDTANPKTPKYK**FVRIQ**PGQTFSVLACYNG-120<br>121-SPSGVYQCAMRPNFTIKG**SFLNG**SCGSVGF-150<br>151-NIDY**DCVSF**CYMHHMELPTGVHAGTDLEGN-180<br>181-FYGPFVDRQTAQAAGTDTTITVNVLAWLYA-210<br>211-AVINGDRWFLNRFTTTLNDFNLVAMKYNYE-240<br>241-PLTQDHVDILGPLSAQTGIAVLDMCASLKE-270<br>271-LLQNGMNGRTILGSALLEDEFTPFDVVRQC-300<br>301-**SGVTFQ**-306	Total sites: 45 (14.70%)<br>Sites within APR: 4 (8.88%)<br>Sites outside APR: 41 (91.12%)
**NSP6**	1-SAVKRTIKGTHHWLLLTILTSLLVL**VQSTQ**-30<br>31-WSLF**FFLYENAFLPF**AMGIIAMSAFAMMFV-60<br>61-KHKHAFLCLFLLPSLA**TVAYFNMV**YMPAS**W**-90<br>91-**VMRI**MTWLDMVDTSLSGFKLKDCVMYASA**V**-120	Total sites: 92 (31.72%)<br>Sites within APR: 30 (32.60%)<br>Sites outside APR: 62 (67.40%)

	121-VLLILMTARTVYDDGARRVWTLMNVLTLVY-150<br>151-KVYYGNALDQAISMWALIISVTSNYSGVVT-180<br>181-TVMFLARGIVFMCVEYCPIFFITGNTLQCI-210<br>211-MLVYCFLGYFCTCYFGLFCLLNRYFRLTLG-240<br>241-VYDYLVSTQEFRYMNSQGLLPPKNSIDAFK-270<br>271-LNIKLLGVGGKPCIKVATVQ-290	
**NSP7**	1-SKMSDVKCTSVVLLSVLQQLRVESSSKLWA-30<br>31-QCVQLHNDILLAKDTTEAFEKMVSLLSVLL-60<br>61-SMQGAVDINKLCEEMLDNRATLQ-83	Total sites: 9 (10.84%)<br>Sites within APR: 1 (11.11%)<br>Sites outside APR: 8 (88.89%)
**NSP8**	1-AIASEFSSLPSYAAFATAQEAYEQAVANGD-30<br>31-SEVVLKKLKKSLNVAKSEFDRDAAMQRKLE-60<br>61-KMADQAMTQMYKQARSEDKRAKVTSAMQTM-90<br>91-LFTMLRKLDNDALNNIINNARDGCVPLNII-120<br>121-PLTTAAKLMVVIPDYNTYKNTCDGTTFTYA-150<br>151-SALWEIQQVVDADSKIVQLSEISMDNSPNL-180<br>181-AWPLIVTALRANSAVKLQ-198	Total sites: 20 (10.10%)<br>Sites within APR: 2 (10.0%)<br>Sites outside APR: 18 (90.0%)
**NSP9**	1-NNELSPVALRQMSCAAGTTQTACTDDNALA-30<br>31-YYNTTKGGRFVLALLSDLQDLKWARFPKSD-60<br>61-GTGTIYTELEPPCRFVTDTPKGPKVKYLYF-90<br>91-IKGLNNLNRGMVLGSLAATVRLQ-113	Total sites: 16 (14.15%)<br>Sites within APR: 0 (0.0%)<br>Sites outside APR: 16 (100%)
**NSP10**	1-AGNATEVPANSTVLSFCAFAVDAAKAYKDY-30<br>31-LASGGQPITNCVKMLCTHTGTGQAITVTPE-60<br>61-ANMDQESFGGASCCLYCRCHIDHPNPKGFC-90<br>91-DLKGKYVQIPTTCANDPVGFTLKNTVCTVC-120<br>121-GMWKGYGCSCDQLREPMLQ-139	Total sites: 17 (12.23%)<br>Sites within APR: 2 (11.76%)<br>Sites outside APR: 15 (88.24%)
**NSP11**	1-SADAQSFLNGFAV-13	Total sites: 2 (15.38%)<br>Sites within APR: 1 (50%)<br>Sites outside APR: 1 (50%)
**NSP12**	1-SADAQSFLNRVCGVSAARLTPCGTGTSTDV-30<br>31-VYRAFDIYNDKVAGFAKFLKTNCCRFQEKD-60<br>61-EDDNLIDSYFVVKRHTFSNYQHEETIYNLL-90<br>91-KDCPAVAKHDFFKFRIDGDMVPHISRQRLT-120<br>121-KYTMADLVYALRHFDEGNCDTLKEILVTYN-150<br>151-CCDDDYFNKKDWYDFVENPDILRVYANLGE-180<br>181-RVRQALLKTVQFCDAMRNAGIVGVLTLDNQ-210<br>211-DLNGNWYDFGDFIQTTPGSGVPVVDSYYSL-240<br>241-LMPILTLTRALTAESHVDTDLTKPYIKWDL-270<br>271-LKYDFTEERLKLFDRYFKYWDQTYHPNCVN-300<br>301-CLDDRCILHCANFNVLFSTVFPPTSFGPLV-330<br>331-RKIFVDGVPFVVSTGYHFRELGVVHNQDVN-360	Total sites: 155 (16.63%)<br>Sites within APR: 21 (13.54%)<br>Sites outside APR: 134 (86.46%)

	361-LHSSRLSFKE**LLVYA**ADPAMHAASGNLLLD-390<br>391-KRTTCFSVAALTNNVAFQTVKPGNFNKDFY-420<br>421-DFAVSKGFFKEGSSVELKHFFFAQDGNAAI-450<br>451-SDYDYYRYNLPTMCDIRQLLFVVEVVDKYF-480<br>481-DCYDGGCINA**NQVIV**NNLDKSAGFPFNKWG-510<br>511-KARLYYDSMSYEDQDALFAYTKRNVIPTIT-540<br>541-QMNLKYAISAKNRARTVA**GVSIC**STMTN**RQ**-570<br>571-**FHQ**KLLKSIAATRG**ATVVIG**TSKFYGGWHN-600<br>601-MLKTVYSDVENPHLMGWDYPKCDRAMPNML-630<br>631-RIMASLVLARKHTTCCSLSHRFYRLANECA-660<br>661-QVLSEMVMCG**GSLYV**KPGGTSSGDATTAYA-690<br>691-N**SVFNIC**QAVTANVNALLSTDGNKIADKYV-720<br>721-RNLQHRLYECLYRNRDVDTDFVNEFYAYLR-750<br>751-KHFSMMILSDDAVVCFNSTYASQG**LVASI**K-780<br>781-NFK**SVLYY**QNNVFMSEAKCWTETDLTKGPH-810<br>811-EFCSQHTMLVKQGDDYV**YLPYP**DPSRILGA-840<br>841-GC**FVDDI**VKTDGTLMIERFVSLAIDAYPLT-870<br>871-KHPNQEYADVFHLYLQYIRKLHD**ELTGH**ML-900<br>901-DMYSVMLTNDNTSRYWEPEFYEAMYTPHTV-930<br>931-LQ-932	
**NSP13**	1-AVGACVLCNSQTSLRCGACIRRPFLCCKCC-30<br>31-YDHVISTSHK**LVLSV**NPYVCNAPGCDVTDV-60<br>61-TQLYLGGMSYYCKSHKPPISFPLCANGQVF-90<br>91-GLYKNTCVGSDNVTDFNAIATCDWTNAGDY-120<br>121-ILANTCTERLKLFAAETLKATEETFKLSYG-150<br>151-IATVREVLSDRELHLSWEVGKPRPPLNRNY-180<br>181-VFTGYRVTKNSKVQIGEYTF**EKGDY**GDAVV-210<br>211-YRGTTTYKLNVGDYFVLTSHTVMPLSAPTL-240<br>241-VPQEHYVRITGLYP**TLNIS**DEFSSN**VANYQ**-270<br>271-KVGMQKYSTLQGPPGTGKSHFAIG**LALYY**P-300<br>301-SARIVYTACSHAAVDALCEKALKYLPIDKC-330<br>331-SRIIPARARVECFDKFKVNSTLEQYVFCTV-360<br>361-NALPETTADIVVFDEISMATNYDLSVVNAR-390<br>391-LRAK**HYVYI**GDPAQLPAPRTLLTKGTLEPE-420<br>421-**YFNSV**CRLMKTIGPDMFLGTCRRCPAEIVD-450<br>451-TVSALVYDNKLKAHKDKSAQCFKMFYK**GVI**-480<br>481-**TH**DVSSAINRPQI**GVVRE**FLTRNPAWRKAV-510<br>511-FISPYNSQNAVASKILGLPTQTVDSSQGSE-540<br>541-Y**DYVIF**TQTTETAHSCNVNRFNVAITRAKV-570<br>571-GILCIMSDRDLYDKLQFTSLEIPRRNVATL-600<br>601-Q	Total sites: 86<br>(14.30%)<br>Sites within APR: 9<br>(10.47%)<br>Sites outside APR: 77<br>(89.53%)
**NSP14**	1-AENVTGLFKDCSKVITGLHPTQAPTHLSVD-30<br>31-TKFKTEG**LCVDI**PGIPKDMTYRRLISMMGF-60<br>61-KMNYQVNGYPNMFITREEAIRHVRAWIGFD-90<br>91-VEGCHATREAVGTNLPLQLGFST**GVNLV**AV-120<br>121-PTGYVDTPNNTDFSRVSAKPPPGDQFKHLI-150<br>151-PLMYKGLPW**NVVRI**KIVQMLSDTLKNLSDR-180	Total sites: 99<br>(18.78%)<br>Sites within APR: 11<br>(11.11%)<br>Sites outside APR: 88<br>(88.89%)

	181-VVFVLWAHGFELTSMKYFVKIGPERTCCLC-210<br>211-DRRATCFSTASDTYACWHHSIGFDYVYNPF-240<br>241-MIDVQQWGFTGNLQSNHDLYCQVHGNAHVA-270<br>271-SCDAIMTRCLAVHECFVKRVDWTIEYPIIG-300<br>301-DELKINAACRKVQHMVVKAALLADKFPVLH-330<br>331-DIGNPKAIKCVPQADVEWKFYDAQPCSDKA-360<br>361-YKIEELFYSYATHSDKFTDGVCLFWNCNVD-390<br>391-RYPANSIVCRFDTRVLSNLNLPGCDGGSLY-420<br>421-VNKHAFHTPAFDKSAFVNLKQLPFFYYSDS-450<br>451-PCESHGKQVVSDIDYVPLKSATCITRCNLG-480<br>481-GAVCRHHANEYRLYLDAYNMMISAGFSLWV-510<br>511-YKQFDTYNLWNTFTRLQ-527	
**NSP15**	1-SLENVAFNVVNKGHFDGQQGEVPVSIINNT-30<br>31-VYTKVDGVDVELFENKTTLPVNVAFELWAK-60<br>61-RNIKPVPEVKILNNLGVDIAANTVIWDYKR-90<br>91-DAPAHISTIGVCSMTDIAKKPTETICAPLT-120<br>121-VFFDGRVDGQVDLFRNARNGVLITEGSVKG-150<br>151-LQPSVGPKQASLNGVTLIGEAVKTQFNYYK-180<br>181-KVDGVVQQLPETYFTQSRNLQEFKPRSQME-210<br>211-IDFLELAMDEFIERYKLEGYAFEHIVYGDF-240<br>241-SHSQLGGLHLLIGLAKRFKESPFELEDFIP-270<br>271-MDSTVKNYFITDAQTGSSKCVCSVIDLLLD-300<br>301-DFVEIIKSQDLSVVSKVVKVTIDYTEISFM-330<br>331-LWCKDGHVETFYPKLQ-346	Total sites: 39 (11.27%)<br>Sites within APR: 9 (23.07%)<br>Sites outside APR: 30 (76.93%)
**NSP16**	1-SSQAWQPGVAMPNLYKMQRMLLEKCDLQNY-30<br>31-GDSATLPKGIMMNVAKYTQLCQYLNTLTLA-60<br>61-VPYNMRVIHFGAGSDKGVAPGTAVLRQWLP-90<br>91-TGTLLVDSDLNDFVSDADSTLIGDCATVHT-120<br>121-ANKWDLIISDMYDPKTKNVTKENDSKEGFF-150<br>151-TYICGFIQQKLALGGSVAIKITEHSWNADL-180<br>181-YKLMGHFAWWTAFVTNVNASSSEAFLIGCN-210<br>211-YLGKPREQIDGYVMHANYIFWRNTNPIQLS-240<br>241-SYSLFDMSKFPLKLRGTAVMSLKEGQINDM-270<br>271-ILSLLSKGRLIIRENNRVVISSDVLVNN-298	Total sites: 44 (14.76%)<br>Sites within APR: 5 (11.36%)<br>Sites outside APR: 39 (88.64%)

Supplementary table 10: Raman band positions of monomeric and aggregated peptides. The positions of amide I bands (1670-1675 for β-sheet and 1660-1682 for disordered), amide II bands (1550-1564 for β-sheet and 1548-1561 for disordered), amide III bands (1220-1230 for β-sheet and 1240-1279 for disordered), and Cα-H bands (1395-1406 for β-sheet and 1380-1390 for disordered) are used as a reference from Ramachandran et al.[39]

Protein/peptide	Monomer				Aggregate			
	Amide I (cm^{-1})	Amide II (cm^{-1})	Amide III (cm^{-1})	Cα−H (cm^{-1})	Amide I (cm^{-1})	Amide II (cm^{-1})	Amide III (cm^{-1})	Cα−H (cm^{-1})

**SP-CoV2**	1668.1	1548.3	1241	1387	1670	1554.2	1228.3	1402.6
**FP1-CoV**	1663	1560	NC	NC	1671.2	1562.5	NC	NC
**FP2-CoV**	1665.9	1551.7	1250	1390.4	1672.2	1556.1	1236.9	1399.3
**FP1-CoV2**	1657.4	1554.5	NC	1389	1672.7	1556	1235	1399
**FP2-CoV2**	1655.8	1554	1229	1385.5	1673.1	1552.2	1232.2	1396
**ORF10**	1665.6	1565	NC	NC	1673.2	1566.2	NC	1405.1

*NC stands for not clear.

Supplementary table 11. Information of peptides and proteins used for *in vitro* aggregation assay.

**Protein and peptide**	**UniProt ID**	**Sequence**	**Solvent**	**Concentration used for *in vitro* aggregation**
**SARS-CoV**				
S fusion peptide 1 (FP1-CoV)	P59594	NH_2-SFIEDLLFNKVTLADAGFMKQY-COOH	Water	1 mg/ml
S fusion peptide 2 (FP2-CoV)	P59594	NH_2-KQYGECLGDINARDLICAQKF-COOH	Water	1 mg/ml
NSP11-CoV	P0C6U8	NH_2-SADASTFLNGFAV-COOH	20 mM Sodium phosphate buffer (pH 7.4)	3 mg/ml
**SARS-CoV-2**				
S signal peptide (SP-CoV2)	P0DTC2	NH_2-MFVFLVLLPLVS-COOH	50% DMSO and 50% 20 mM phosphate buffer (pH 7.4)	0.5 mg/ml

S fusion peptide 1 (FP1-CoV2)	P0DTC2	NH_2-SFIEDLLFNKVTLADAGFIKQY-COOH	50% DMSO and 50% 20 mM phosphate buffer (pH 7.4)	1 mg/ml
S fusion peptide 2 (FP2-CoV2)	P0DTC2	NH_2-KQYGDCLGDIAARDLICAQKF-COOH	10% DMSO and 90% 20 mM phosphate buffer (pH 7.4)	1 mg/ml
ORF10 (full-length protein)	YP_009725255.1	NH_2-MGYINVFAFPFTIYSLLLCRMNSRNYIAQVDVVNFNLT-COOH	60% DMSO and 40% 20 mM phosphate buffer (pH 7.4)	1 mg/ml
NSP6-p (Residues 91-112 of NSP6)	P0DTC1	NH_2-VMRIMTWLDMVDTSLSGFKLKD-COOH	8% DMSO and 92% 20 mM phosphate buffer (pH 7.4	1 mg/ml
NSP11-CoV2 (full-length)	P0DTC1	NH_2-SADAQSFLNGFAV-COOH	20 mM phosphate buffer (pH 7.4) containing 50 mM NaCl	3 mg/ml

Supplementary table 12. Information of peptides given in mass spectrometry data in Supplementary Dataset.

**Name of the peptide**	**Sample name**	**Sample ID**	**Purity**	**Molecular Weight (Da)**
SP-CoV2	CV2_Signal Peptide	U1200FK090-6	92.6%	1377.78
FP1-CoV	CoV1_S_FP1	U083MGG030-1	97.5%	2550.93
FP2-CoV	CoV1_S_FP2	U083MGG030-3	90.7%	2385.73
FP1-CoV2	Fusion Peptide 1	U1200FK090-3	79%	2532.90
FP2-CoV2	Fusion Peptide 2	U1200FK090-5	Crude	2328.68
ORF10	Cov_ORF10	A3893-6	Crude	4449.24
NSP6-p	NSP6_90-112	U521TFI050-14	88.5%	2587.10
NSP11-CoV	NSP11_CoV1	U083MGG030-5	92.9%	1299.39
NSP11-CoV2	NSP11	U4944FD210-1	72.9%	1326.42

Supplementary Figures:

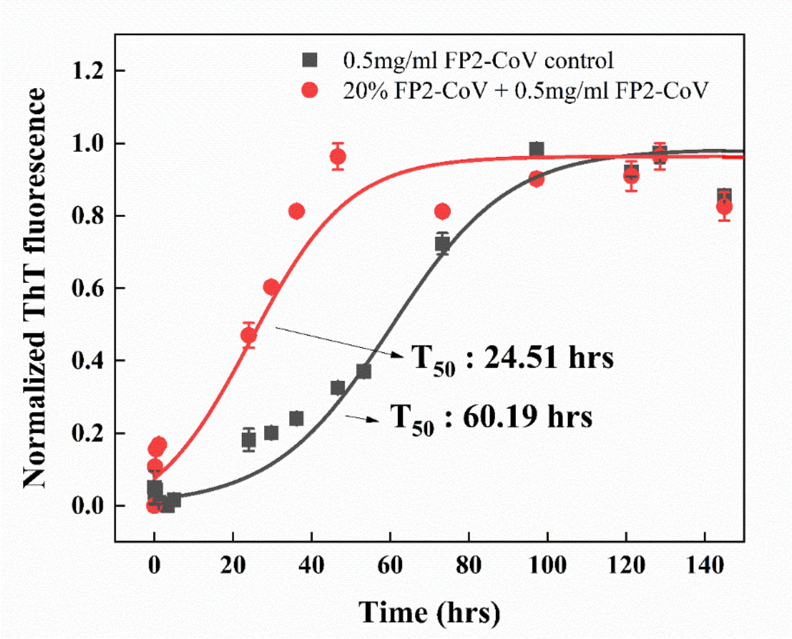

Supplementary figure 1. Self-seeding activity of FP2-CoV peptide aggregates. At 0.5 mg/ml concentration, the FP2-CoV peptide aggregation process has a T_{50} value of 60.19 ± NC hrs (at 1 mg/ml concentration, T_{50} is 47.64 ± 3.75 hrs, as reported in the main manuscript). Upon addition of 20% FP2-CoV seeds (sonicated preformed amyloid fibrils), its T_{50} decreases to 24.51 ± NC hrs. The kinetics reactions are monitored using ThT fluorescence intensity at 490 nm, where dot (FP2-CoV + FP2-CoV seeds) and square symbols (FP2-CoV control) indicate the average value of two technical replicates and the error bar represents SEM of these replicates. The black- and red-coloured lines indicate the sigmoidal fittings of data points which determines the T_{50} values. These results show that the seeds catalyze the aggregation of peptides into amyloids. NC stands for not calculated. The source data are given in the Source Data file.

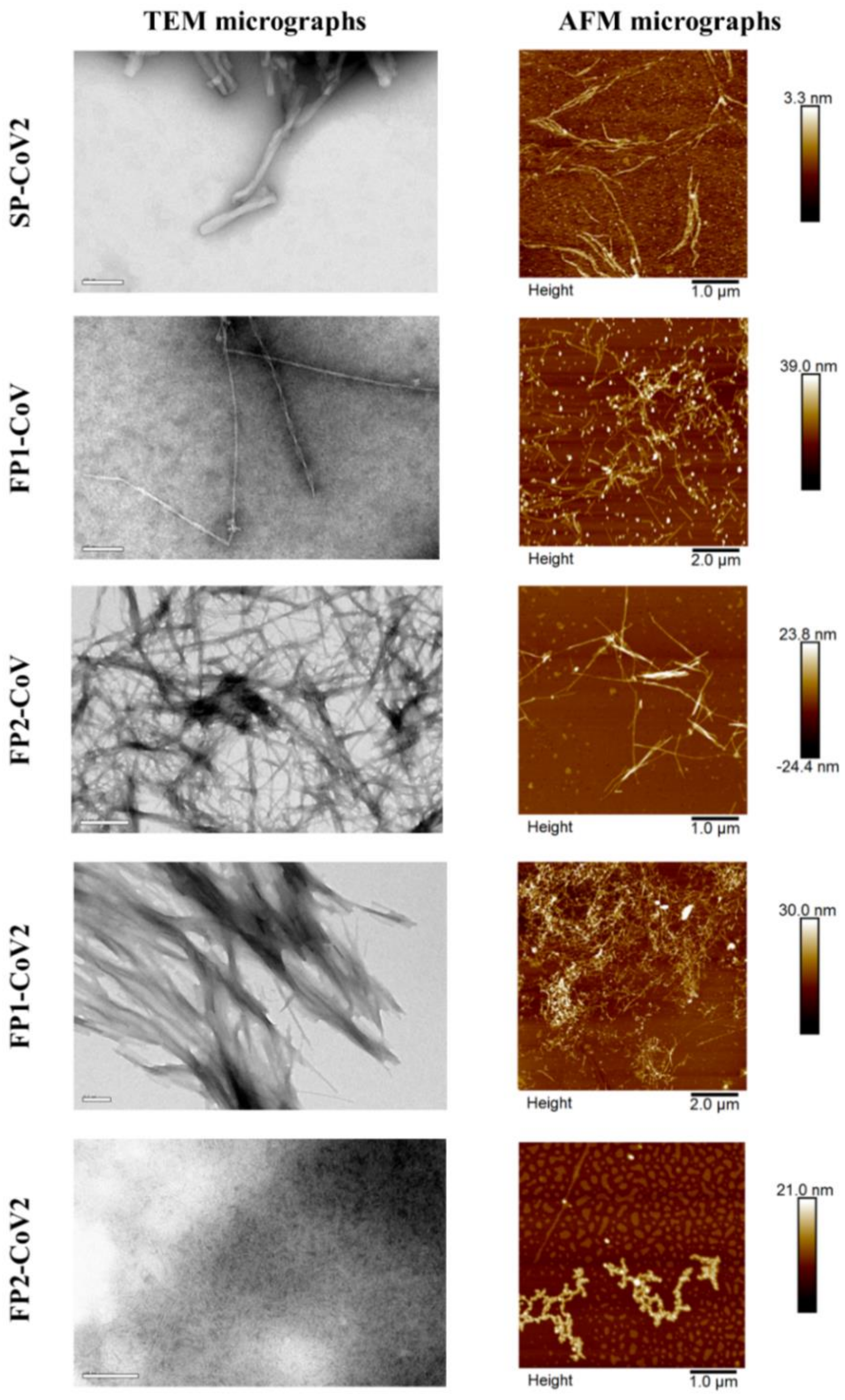

Supplementary figure 2. TEM and AFM micrographs of SARS-CoV and SARS-CoV-2 peptides. For TEM images, the scale bar represents 100 nm for SP-CoV2, FP1-CoV, and FP2-CoV2, 500 nm for FP2-CoV, and 200 nm for FP1-CoV2 peptide aggregates, respectively.

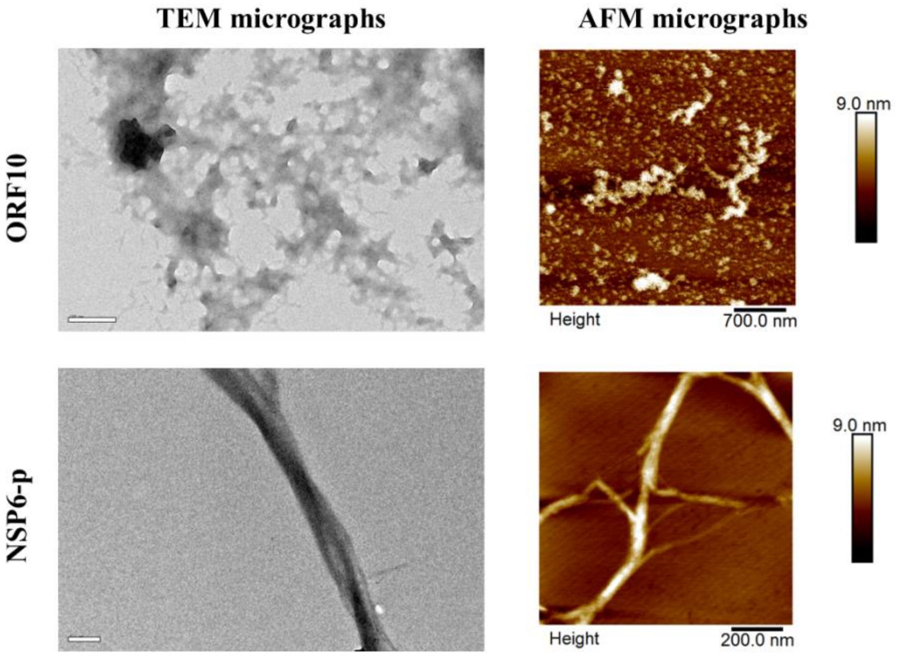

Supplementary figure 3. TEM and AFM micrographs of SARS-CoV-2 peptides. For TEM images, the scale bar represents 200 nm for ORF10 and NSP6-p peptide aggregates.

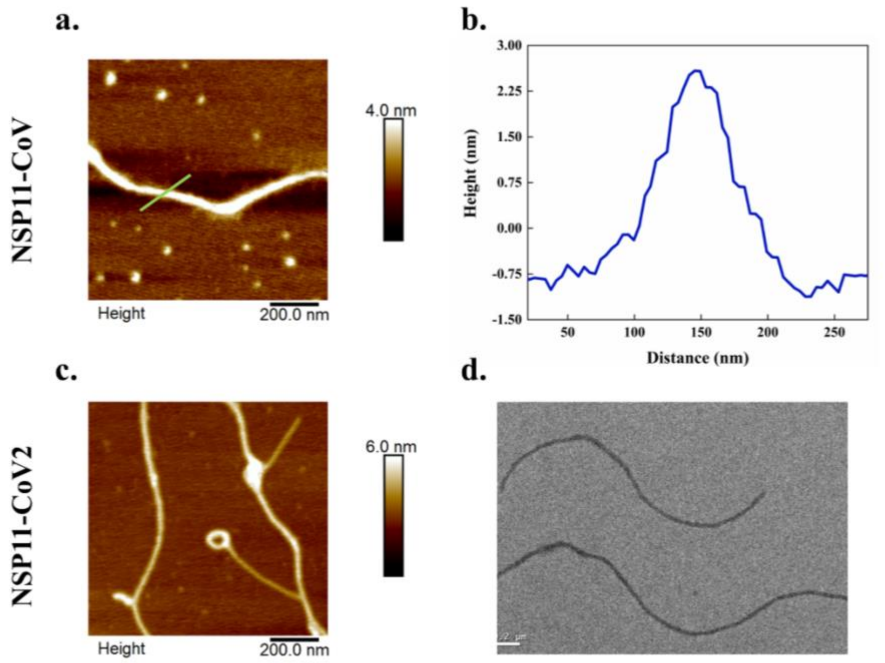

Supplementary figure 4. (a) AFM image of NSP11-CoV fibril. **(b)** Height profile of NSP11-CoV fibril shown with a green-colored line in panel **a**. **(c)** AFM image of NSP11-CoV2 fibrils. **(d)** HR-TEM micrograph of NSP11-CoV2; scale bar represents 200 nm. The source data are given in the Source Data file.

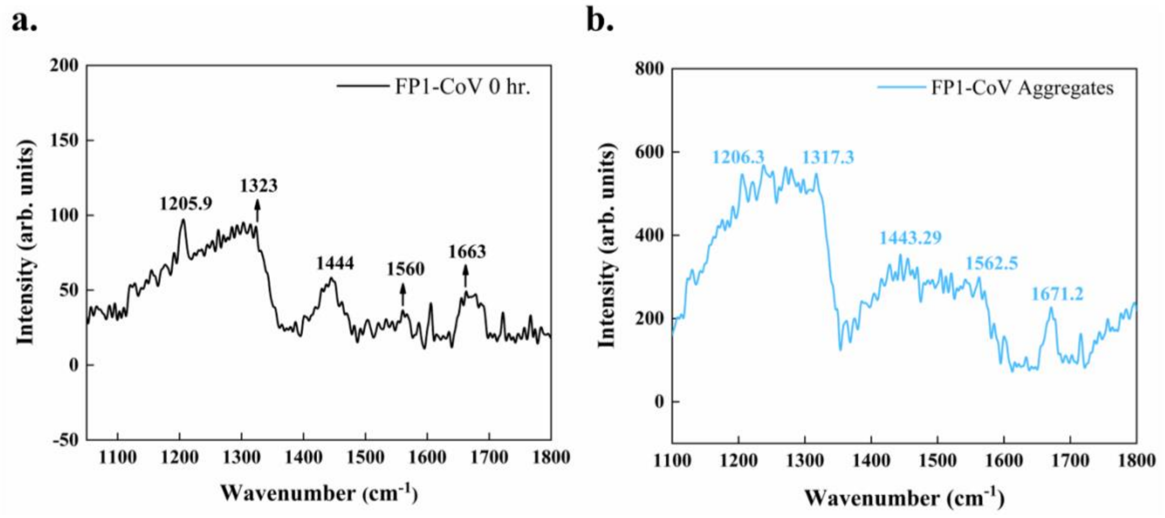

Supplementary figure 5. Raman spectra of monomer **(a)** and aggregates **(b)** of FP1-CoV. The spectral data was smoothened with 10 points using the FFT filter function. The source data are given in the Source Data file. Arb. units are arbitrary units.
