# Supplementary material for: Amyloidogenic proteins in the SARS-CoV and SARS-CoV-2 proteomes
Source: Nat Commun. 2023 Feb 20;14:945. doi: 10.1038/s41467-023-36234-4 (PMC9940680; doi:10.1038/s41467-023-36234-4)
Supplement: Supplementary file 3 — Description for Additional Supplementary Files [file 41467_2023_36234_MOESM3_ESM.pdf]

Description of Additional Supplementary Files

Supplementary Data 1

- Mass spectrometry data of all peptides
