# Supplementary material for: Amyloidogenic proteins in the SARS-CoV and SARS-CoV-2 proteomes
Source: Nat Commun. 2023 Feb 20;14:945. doi: 10.1038/s41467-023-36234-4 (PMC9940680; doi:10.1038/s41467-023-36234-4)
Supplement: Supplementary file 4 — Supplmentary Dataset [file 41467_2023_36234_MOESM4_ESM.pdf]

Sample Name :CV2_Signal Peptide
Sample ID :U1200FK090-6
Time Processed :1:40:56 PM
Month-Day-Year Processed :12/26/2020

Pump A : 0.065% trifluoroacetic in 100% water (v/v)
Pump B : 0.05% trifluoroacetic in 100% acetonitrile (v/v)
Total Flow:1 ml/min
Wavelength:220 nm

Time	Module	Command	Value
0.01	Pumps	B.Conc	5
25.00	Pumps	B.Conc	65
25.01	Pumps	B.Conc	95
26.00	Pumps	B.Conc	95
27.01	Pumps	B.Conc	5
35.00	Controller	Stop	

<<Column Performance>>
<Detector A>
Column : Inertsil ODS-3 4.6 x 250 mm

Chromatogram

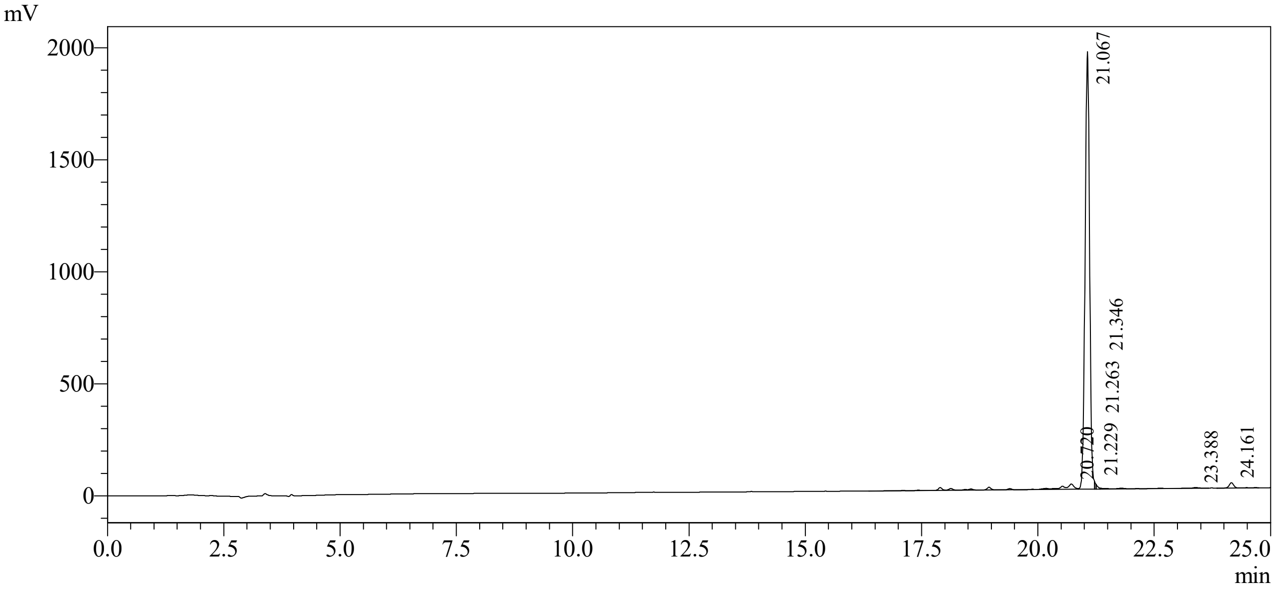

1 Detector A Channel 1 / 220nm

Peak Table

Detector A Channel 1 220nm

Peak#	Ret. Time	Area	Height	Area %
1	20.720	675983	23657	4.759
2	21.067	13160527	1952551	92.643
3	21.229	51728	30595	0.364
4	21.263	48912	18384	0.344
5	21.346	81610	4022	0.574
6	23.388	33037	3120	0.233
7	24.161	153773	23290	1.082
Total		14205570	2055619	100.000

Mass Spectrum

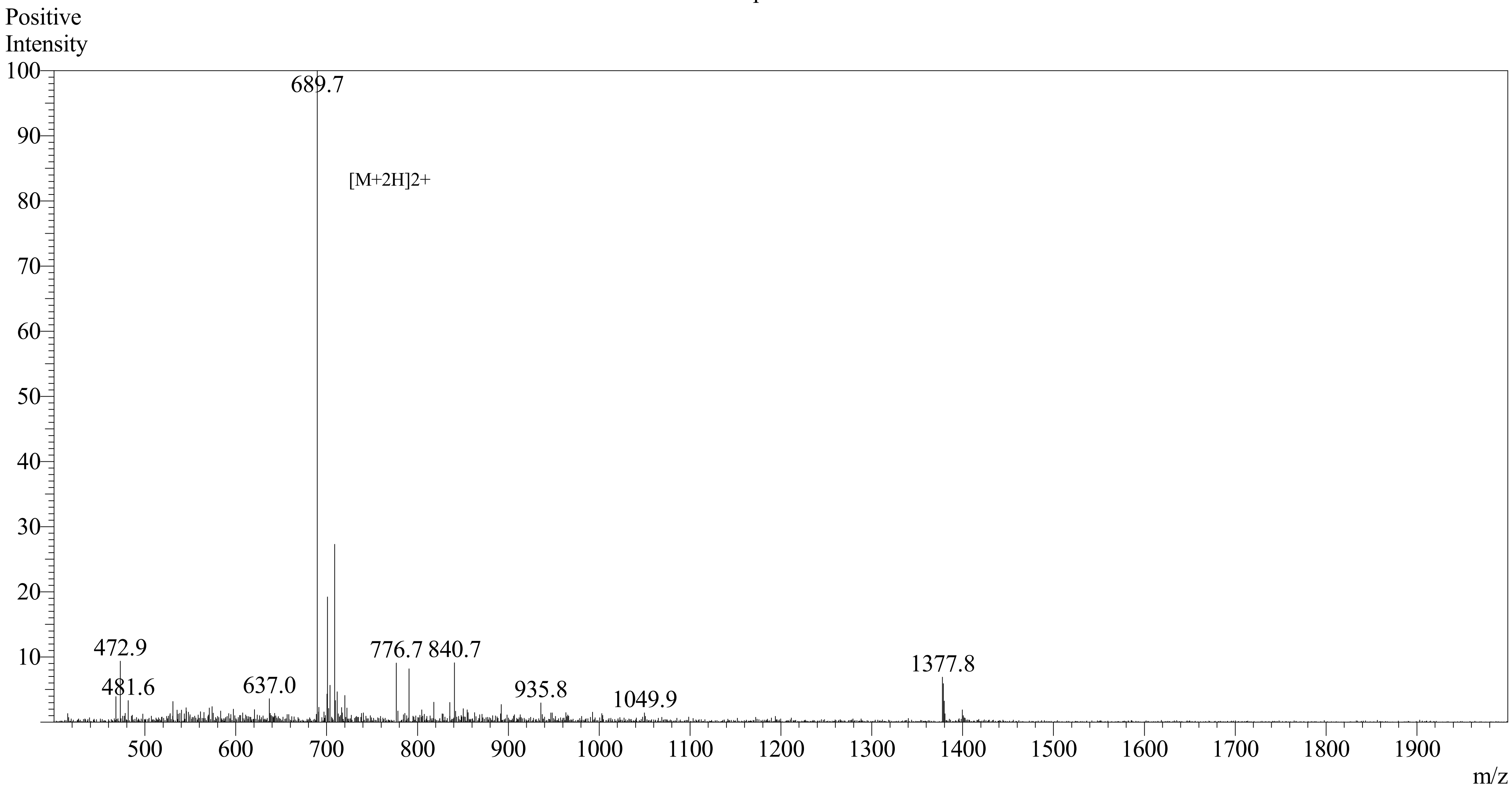

Sample Information	
Month-Day Processed :	12/26/20
Time Processed :	3:37:21 PM
Injection Volume :	0.4
Sample Name :	CV2_Signal Peptide
Sample ID :	U1200FK090-6
Theoretical MW :	1377.78
Observed MW :	1377.4

Interface	:ESI
Nebulizing Gas Flow	:1.5L/min
CDL Temp	:250
Block Temp	:200

Equipment	: GK11010007
Interface Bias	: +4.5 kV
Drying Gas Flow	:5 L/min
T.Flow	:0.2 ml/min
B.conc	:50%H2O/50%MeOH

Sample Name :CoV1_S_FP1
Sample ID :U083MGG030-1
Time Processed :2:07:07 AM
Month-Day-Year Processed :09/07/2021

Pump A : 0.065% trifluoroacetic in 100% water (v/v)
Pump B : 0.05% trifluoroacetic in 100% acetonitrile (v/v)
Total Flow:1 ml/min
Wavelength:220 nm
<<LC Time Program>>

Time	Module	Command	Value
0.01	Pumps	B.Conc	5
25.00	Pumps	B.Conc	65
25.01	Pumps	B.Conc	95
27.00	Pumps	B.Conc	95
27.01	Pumps	B.Conc	5
35.00	Pumps	B.Conc	5
35.01	Controller	Stop	

<<Column Performance>>
<Detector A>
Column :Inertsil ODS-3 4.6 x 250 mm
Equipment: ZJ21010026

<Chromatogram>

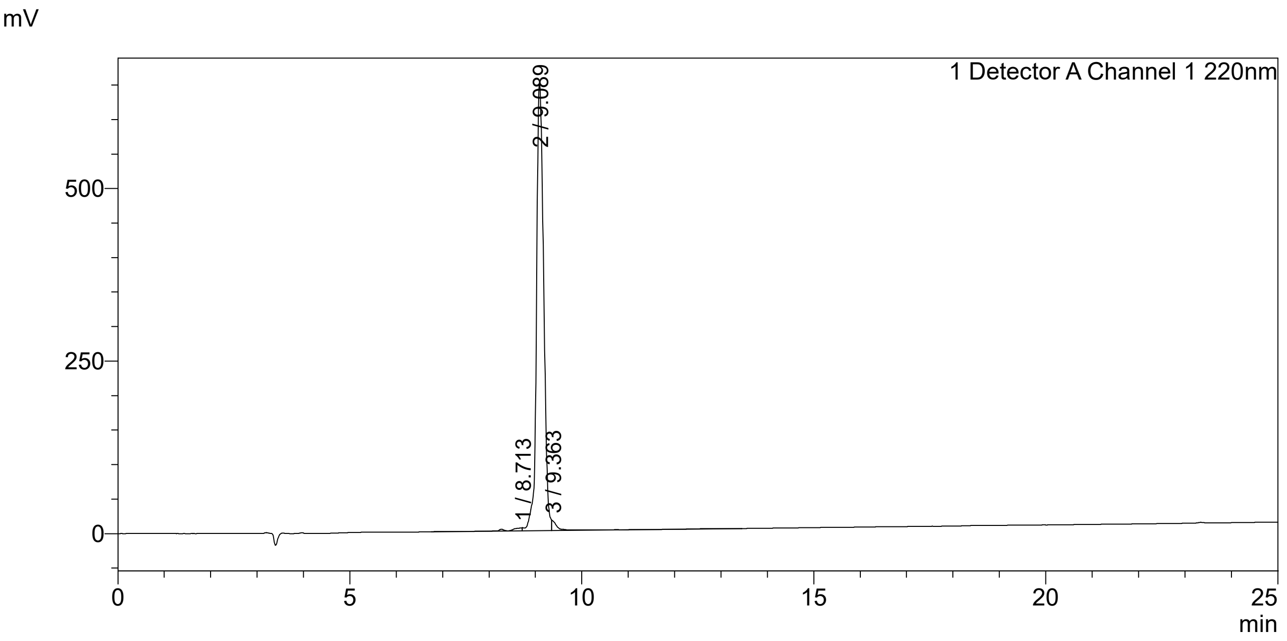

<Peak Table>

Detector A Channel 1 220nm

Peak#	Ret. Time	Area	Height	Area%
1	8.713	67324	4305	0.946
2	9.089	6933708	647309	97.456
3	9.363	113673	14864	1.598
Total		7114705	666479	100.000

Sample Name :CoV1_S_FP2
Sample ID :U083MGG030-3
Time Processed :9:53:08 AM
Month-Day-Year Processed :08/30/2021

Pump A : 0.065% trifluoroacetic in 100% water (v/v)
Pump B : 0.05% trifluoroacetic in 100% acetonitrile (v/v)
Total Flow:1 ml/min
Wavelength:220 nm
<<LC Time Program>>

Time	Module	Command	Value
0.01	Pumps	B.Conc	5
25.00	Pumps	B.Conc	65
25.01	Pumps	B.Conc	95
27.00	Pumps	B.Conc	95
27.01	Pumps	B.Conc	5
35.00	Pumps	B.Conc	5
35.01	Controller	Stop	

<<Column Performance>>
<Detector A>
Column :Inertsil ODS-3 4.6 x 250 mm
Equipment: ZJ21010026

<Chromatogram>

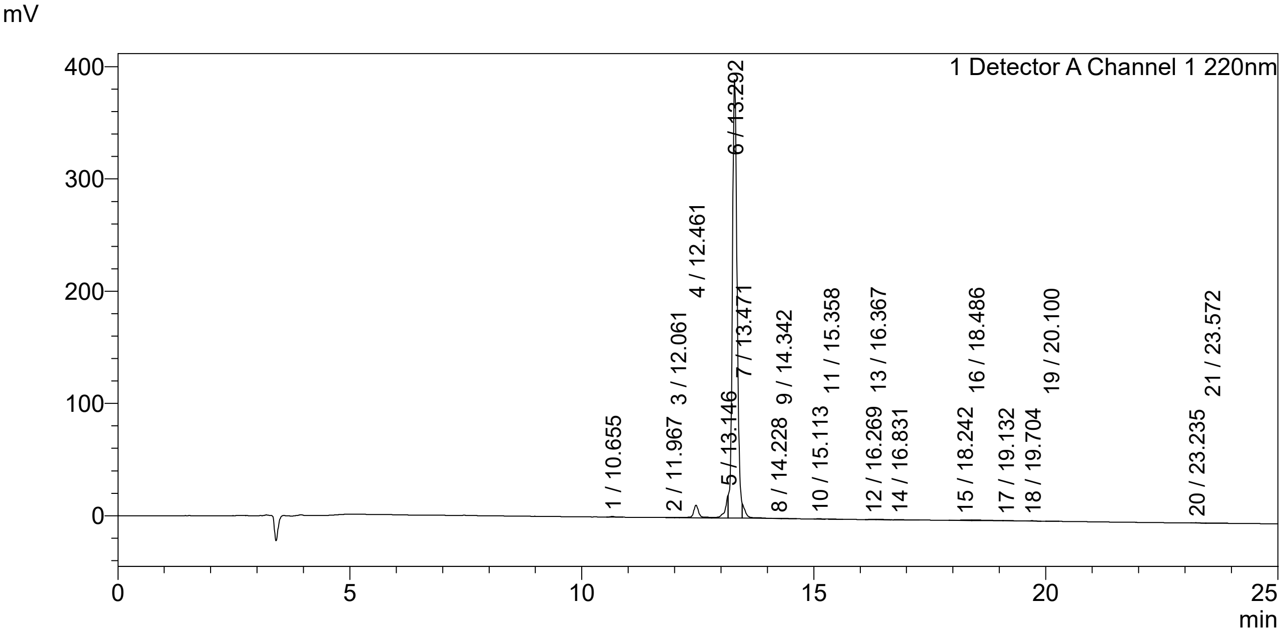

<Peak Table>

Detector A Channel 1 220nm

Peak#	Ret. Time	Area	Height	Area%
1	10.655	4016	588	0.130
2	11.967	1298	150	0.042
3	12.061	1125	204	0.037
4	12.461	90028	11203	2.922
5	13.146	87896	19076	2.852
6	13.292	2795913	390881	90.732
7	13.471	60288	12105	1.956
8	14.228	2004	248	0.065
9	14.342	2307	254	0.075
10	15.113	3469	440	0.113
11	15.358	1852	171	0.060
12	16.269	2615	277	0.085
13	16.367	3665	302	0.119
14	16.831	2134	122	0.069
15	18.242	6856	346	0.222
16	18.486	7927	421	0.257

Peak#	Ret. Time	Area	Height	Area%
17	19.132	1170	89	0.038
18	19.704	2703	374	0.088
19	20.100	1211	161	0.039
20	23.235	1896	272	0.062
21	23.572	1129	111	0.037
Total		3081506	437794	100.000

Mass Spectrum

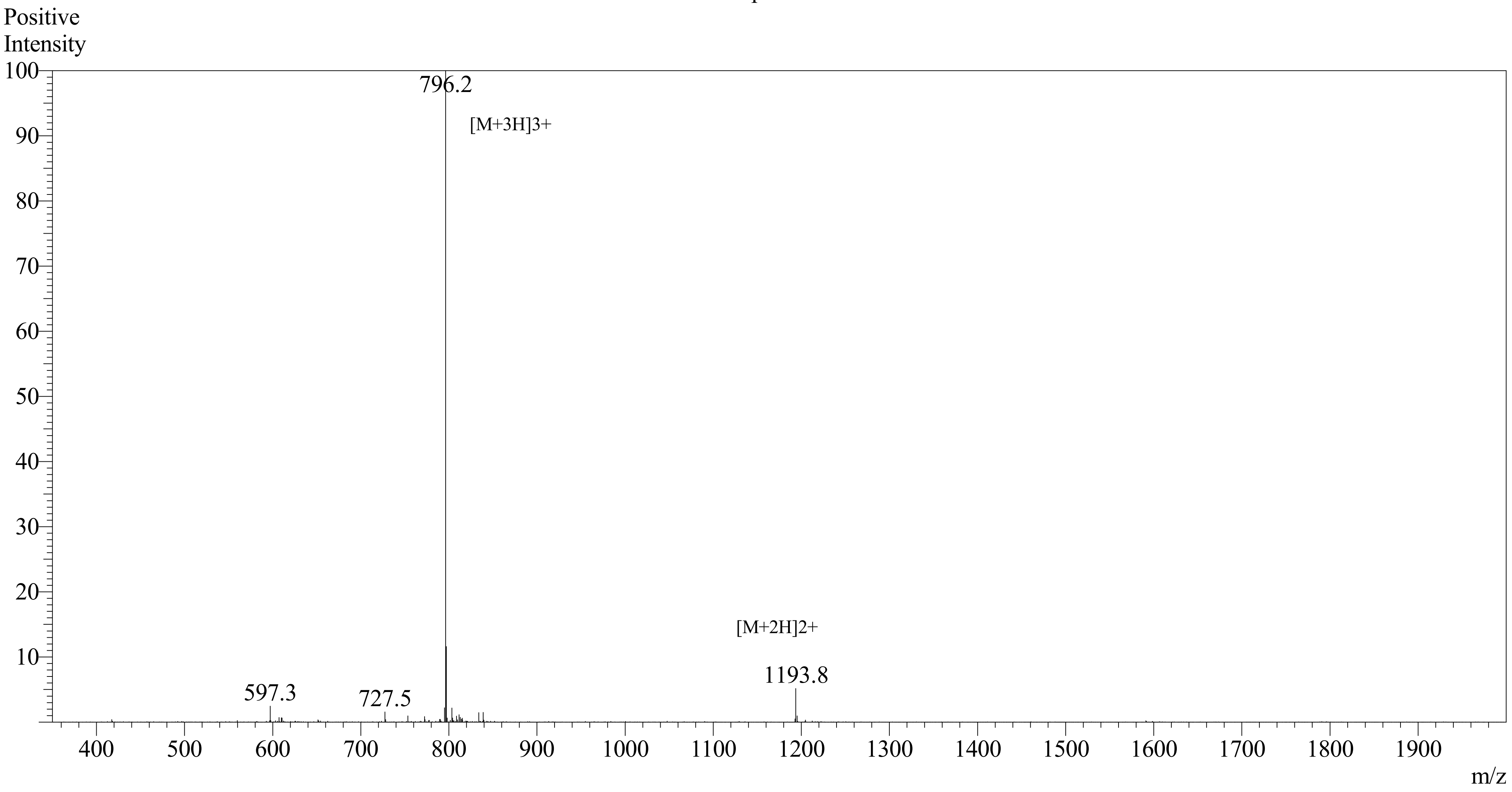

Sample Information	
Month-Day Processed :	08/30/21
Time Processed :	10:23:21
Injection Volume :	0.2
Sample Name :	CoV1_S_FP2
Sample ID :	U083MGG030-3
Theoretical MW :	2385.73
Observed MW :	2385.6

Interface	:ESI
Nebulizing Gas Flow	:1.5L/min
CDL Temp	:250
Block Temp	:200

Equipment	: ZJ21010035
Interface Bias	: +4.5 kV
Drying Gas Flow	:5 L/min
T.Flow	:0.2 ml/min
B.conc	:50%H2O/50%MeOH

Sample Name : Fusion Peptide 1
Sample ID :U1200FK090-3
Time Processed: 5:45:03
Year-Month-Day: 2020/12/22

Pump A : 0.065% trifluoroacetic in 100% water (v/v)
Pump B : 0.05% trifluoroacetic in 100% acetonitrile (v/v)
Total Flow:1 ml/min
<<Detector A>>
Wavelength Ch1 : 220 nm

<<LC Time Program>>

Time	Module	Command	Value	Comment
0.01	Pumps	Solvent B Conc.	5	
25.00	Pumps	Solvent B Conc.	65	
25.01	Pumps	Solvent B Conc.	95	
27.00	Pumps	Solvent B Conc.	95	
27.01	Pumps	Solvent B Conc.	5	
33.00	Pumps	Solvent B Conc.	5	
33.01	Controller	Stop		

<<Column Performance>>
<Detector A>
Column : Inertsil ODS-3 4.6 x 250 mm
Equipment:ZJ19010015

<Chromatogram>

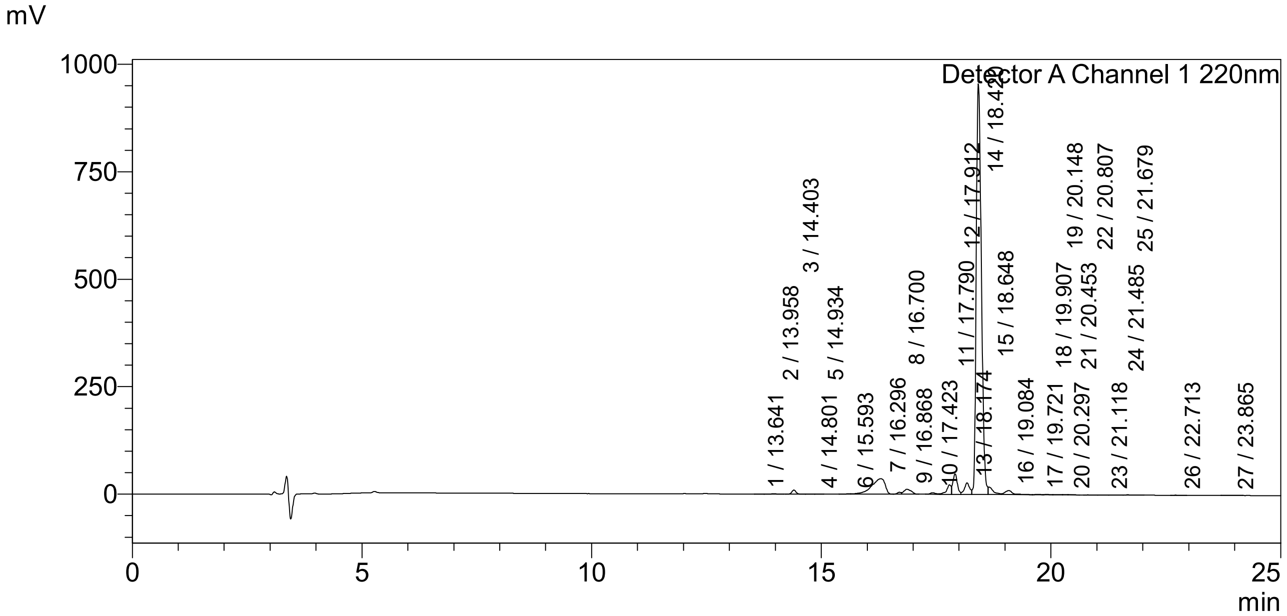

<Peak Table>

Detector A Channel 1 220nm

Peak#	Ret. Time	Area	Height	Area%
1	13.641	549	71	0.006
2	13.958	5769	573	0.062
3	14.403	59162	9554	0.633
4	14.801	1242	174	0.013
5	14.934	1901	263	0.020
6	15.593	6414	792	0.069
7	16.296	739070	35992	7.911
8	16.700	27938	4589	0.299
9	16.868	130079	11559	1.392
10	17.423	31077	3665	0.333
11	17.790	150201	22176	1.608
12	17.912	322498	47775	3.452
13	18.174	214324	26783	2.294
14	18.420	7383210	955236	79.030

Peak#	Ret. Time	Area	Height	Area%
15	18.648	127048	17223	1.360
16	19.084	107560	9371	1.151
17	19.721	5330	874	0.057
18	19.907	3602	610	0.039
19	20.148	1669	196	0.018
20	20.297	1323	194	0.014
21	20.453	3525	451	0.038
22	20.807	1712	276	0.018
23	21.118	1211	118	0.013
24	21.485	5821	399	0.062
25	21.679	6498	570	0.070
26	22.713	1989	266	0.021
27	23.865	1518	139	0.016
Total		9342241	1149889	100.000

Mass Spectrum

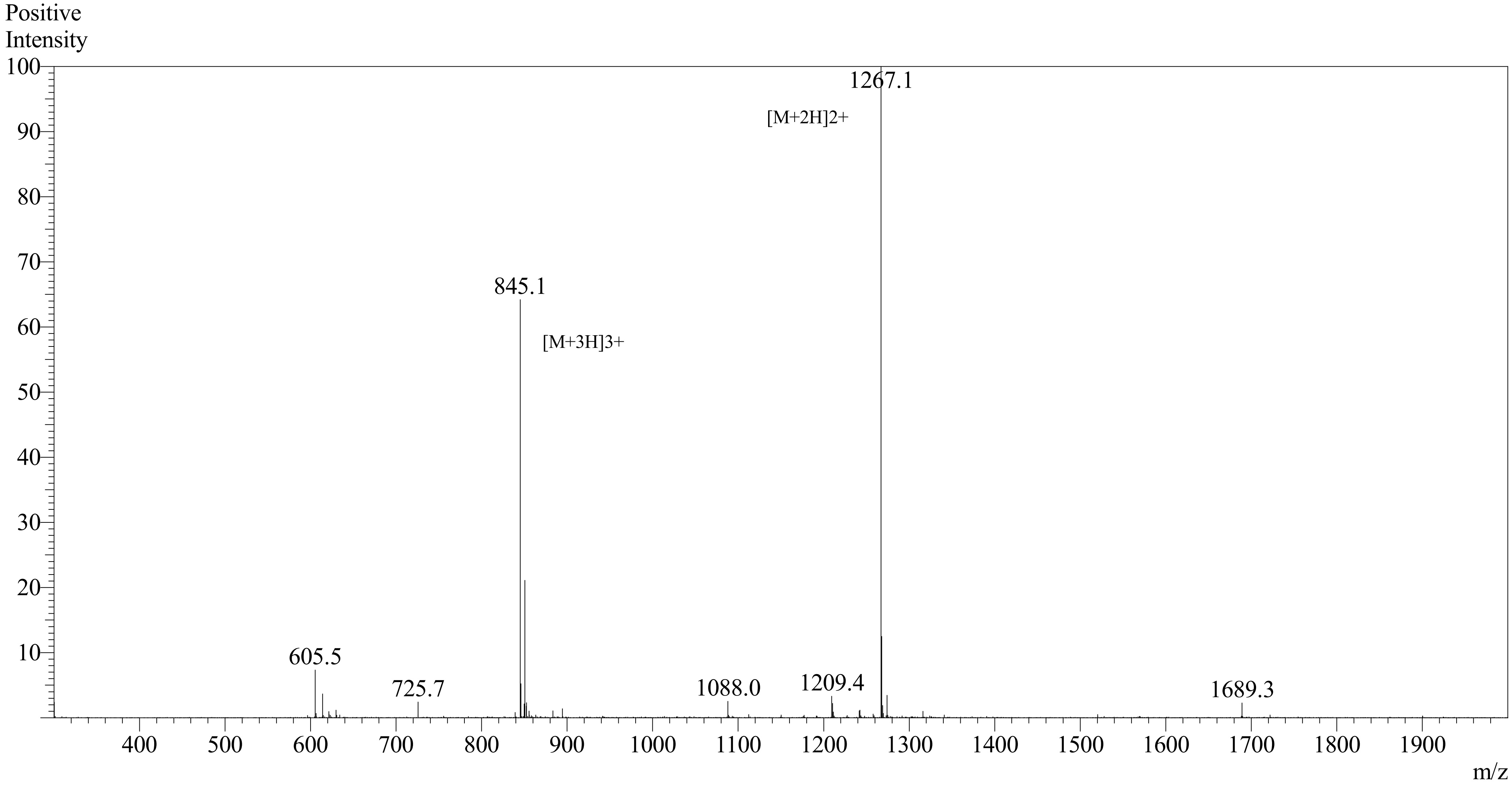

Sample Information	
Month-Day Processed :	12/21/20
Time Processed :	8:15:42 PM
Injection Volume :	0.4
Sample Name :	Fusion Peptide 1
Sample ID :	U1200FK090-3
Theoretical MW :	2532.90
Observed MW :	2532.2

Interface	:ESI
Nebulizing Gas Flow	:1.5L/min
CDL Temp	:250
Block Temp	:200

Equipment	: GK11010007
Interface Bias	: +4.5 kV
Drying Gas Flow	:5 L/min
T.Flow	:0.2 ml/min
B.conc	:50%H2O/50%MeOH

Sample Name :Fusion Peptide 2
Sample ID :U1200FK090-5
Time Processed :1:23:04 AM
Month-Day-Year Processed :12/05/2020

Pump A : 0.065% trifluoroacetic in 100% water (v/v)
Pump B : 0.05% trifluoroacetic in 100% acetonitrile (v/v)
Total Flow:1 ml/min
Wavelength:220 nm
<<LC Time Program>>

Time	Module	Command	Value
0.01	Pumps	B.Conc	5
25.00	Pumps	B.Conc	65
25.01	Pumps	B.Conc	95
27.00	Pumps	B.Conc	95
27.01	Pumps	B.Conc	5
35.00	Pumps	B.Conc	5
35.00	Controller	Stop	

<<Column Performance>>
<Detector A>
Column :Inertsil ODS-3 4.6 x 250 mm
Equipment: ZJ19010324

<Chromatogram>

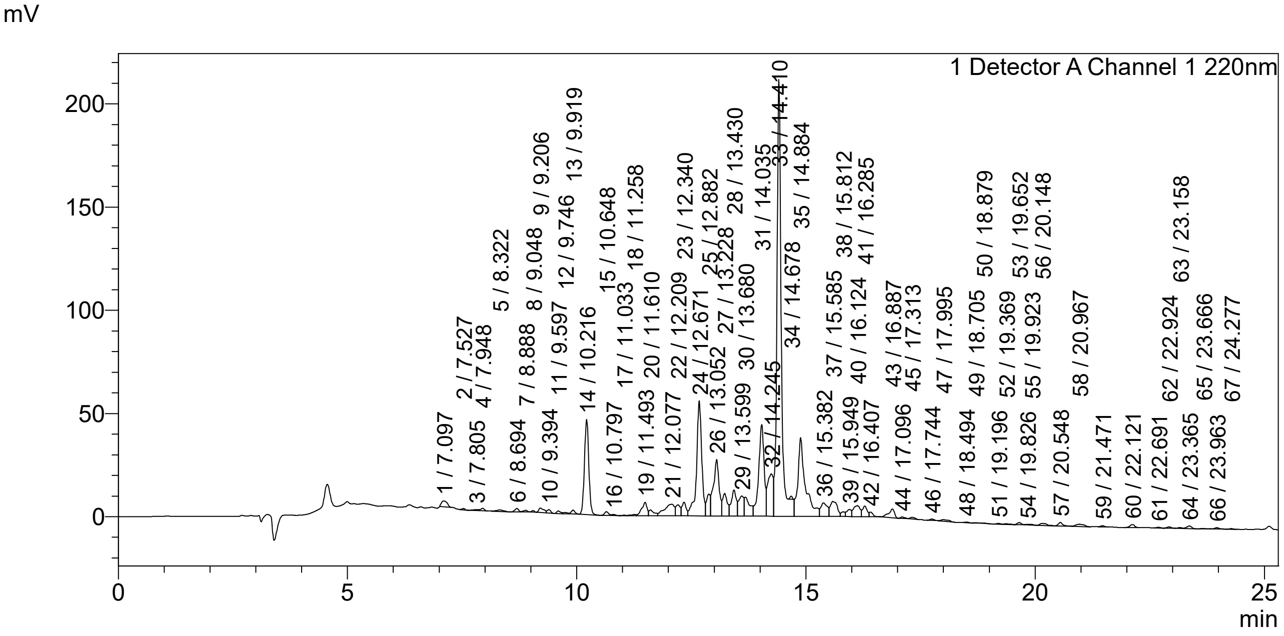

<Peak Table>

Detector A Channel 1 220nm

Peak#	Ret. Time	Area	Height	Area%
1	7.097	25071	2866	0.549
2	7.527	2759	640	0.060
3	7.805	4382	508	0.096
4	7.948	7114	1299	0.156
5	8.322	9436	823	0.206
6	8.694	10297	1739	0.225
7	8.888	5162	819	0.113
8	9.048	3271	753	0.072
9	9.206	17137	2157	0.375
10	9.394	9263	1655	0.203
11	9.597	7804	1281	0.171
12	9.746	4343	611	0.095
13	9.919	11151	1921	0.244
14	10.216	306857	46106	6.715
15	10.648	11130	1835	0.244
16	10.797	2645	479	0.058

Peak#	Ret. Time	Area	Height	Area%
17	11.033	1075	212	0.024
18	11.258	4443	569	0.097
19	11.493	52199	6473	1.142
20	11.610	21771	2789	0.476
21	12.077	86378	5624	1.890
22	12.209	35365	5335	0.774
23	12.340	42018	6583	0.920
24	12.671	445420	55747	9.747
25	12.882	60282	10572	1.319
26	13.052	239662	27373	5.245
27	13.228	78111	10951	1.709
28	13.430	97129	12619	2.126
29	13.599	74494	9732	1.630
30	13.680	78610	9146	1.720
31	14.035	366917	44330	8.029
32	14.245	173797	20473	3.803
33	14.410	1393324	211646	30.491
34	14.678	5388	1544	0.118
35	14.884	436584	38086	9.554
36	15.382	65499	6568	1.433
37	15.585	78243	7283	1.712
38	15.812	13498	2288	0.295
39	15.949	24610	3419	0.539
40	16.124	53929	5306	1.180
41	16.285	36565	5136	0.800
42	16.407	11502	2214	0.252
43	16.887	37903	4330	0.829
44	17.096	4476	708	0.098
45	17.313	5687	784	0.124
46	17.744	4244	674	0.093
47	17.995	9268	743	0.203
48	18.494	2197	353	0.048
49	18.705	1037	182	0.023
50	18.879	1386	186	0.030
51	19.196	2589	390	0.057
52	19.369	2654	336	0.058
53	19.652	5724	983	0.125
54	19.826	1376	275	0.030
55	19.923	1525	326	0.033
56	20.148	10713	966	0.234
57	20.548	10939	1684	0.239
58	20.967	12977	1077	0.284
59	21.471	3559	591	0.078
60	22.121	10865	1497	0.238
61	22.691	2196	363	0.048
62	22.924	4003	620	0.088
63	23.158	2487	398	0.054
64	23.365	9370	1260	0.205
65	23.666	1020	134	0.022
66	23.963	4921	677	0.108
67	24.277	1900	291	0.042
Total		4569652	597339	100.000

Mass Spectrum

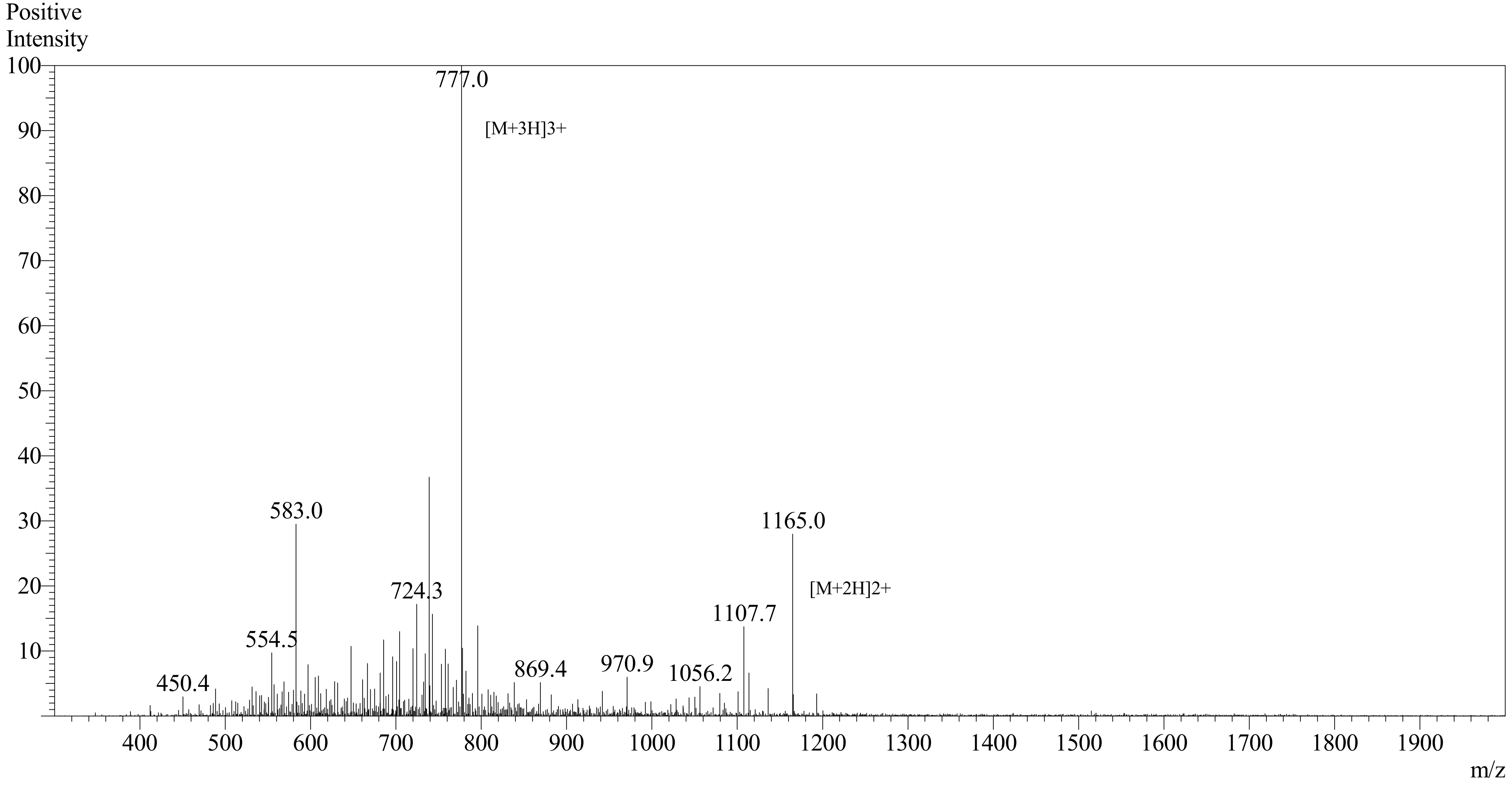

Sample Information	
Month-Day Processed :	12/11/20
Time Processed :	5:15:54 PM
Injection Volume :	0.4
Sample Name :	Fusion Peptide 2
Sample ID :	U1200FK090-5
Theoretical MW :	2328.68
Observed MW :	2328.0

Interface	:ESI
Nebulizing Gas Flow	:1.5L/min
CDL Temp	:250
Block Temp	:200

Equipment	: GK11010007
Interface Bias	: +4.5 kV
Drying Gas Flow	:5 L/min
T.Flow	:0.2 ml/min
B.conc	:50%H2O/50%MeOH

Applied Biosystems Voyager System 1150

Voyager Spec #1=>AdvBC(2,0.5,0.1)=>NR(22.00)=>AdvBC(2,0.5,0.1)[BP = 4449.2, 3839]

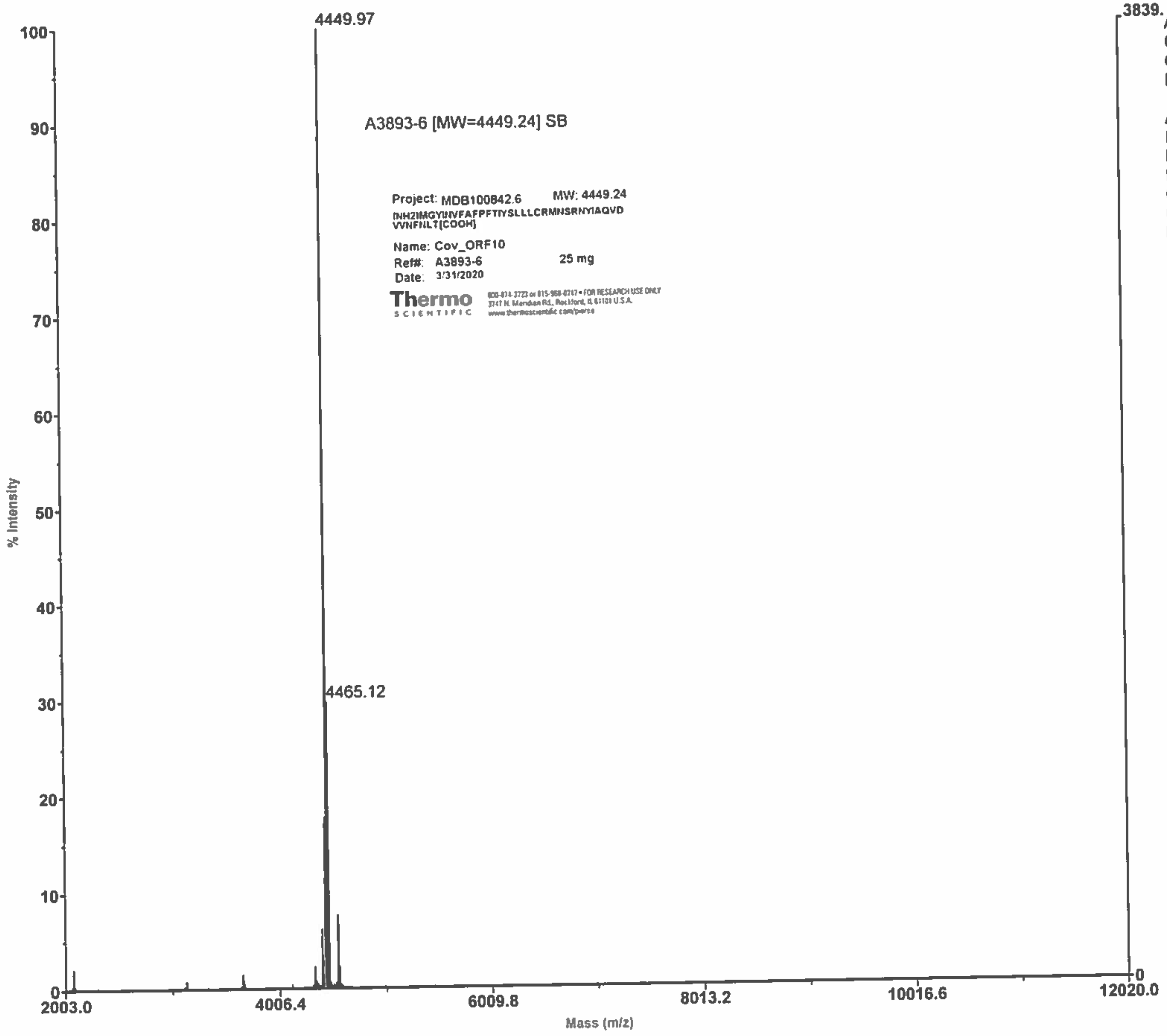

Mode of operation:	Linear
Extraction mode:	Delayed
Polarity:	Negative
Acquisition control:	Manual
Accelerating voltage:	20000 V
Grid voltage:	94%
Guide wire 0:	0.05%
Extraction delay time:	500 nsec
Acquisition mass range:	2000 – 12000 Da
Number of laser shots:	100/spectrum
Laser intensity:	2487
Laser Rep Rate:	3.0 Hz
Calibration type:	Default
Calibration matrix:	a-Cyano-4-hydroxycinnamic acic
Low mass gate:	Off
Digitizer start time:	28.428
Bin size:	2 nsec
Number of data points:	20500
Vertical scale:	200 mV
Vertical offset:	0%
Input bandwidth:	150 MHz
Sample well:	24
Plate ID:	100 WELL PLATE
Serial number:	1150
Instrument name:	Voyager-DE
Plate type filename:	C:\VOYAGER\100 well plate.plt
Lab name:	PE Biosystems
Absolute x-position:	16821.4
Absolute y-position:	35822.2
Relative x-position:	-6.07651
Relative y-posititon:	-1325.32
Shots in spectrum:	12
Source pressure:	1.66e-007
Mirror pressure:	0
TC2 pressure:	0.01982
TIS gate width:	30
TIS flight length:	940

Acquired: 16:12:00, March 13, 2020

Printed: 12:09, March 16, 2020

Sample Name :NSP6_90-112 **Please read 90-112 as 91-112.**
Sample ID :U521TFI050-14
Time Processed: 2:28:28 PM
Month-Day-Year Processed :11/13/2020

Pump A : 0.065% trifluoroacetic in 100% water (v/v)
Pump B : 0.05% trifluoroacetic in 100% acetonitrile (v/v)
Total Flow:1 ml/min
Wavelength:220 nm
<<LC Time Program>>

Time	Module	Command	Value
0.01	Pumps	Solvent B Conc.	5
25.00	Pumps	Solvent B Conc.	65
25.01	Pumps	Solvent B Conc.	95
27.00	Pumps	Solvent B Conc.	95
27.01	Pumps	Solvent B Conc.	5
33.00	Pumps	Solvent B Conc.	5
33.00	Controller	Stop	

<<Column Performance>>
<Detector A>
Column : Inertsil ODS-3 4.6 x 250 mm
Equipment:ZJ19010325

<Chromatogram>

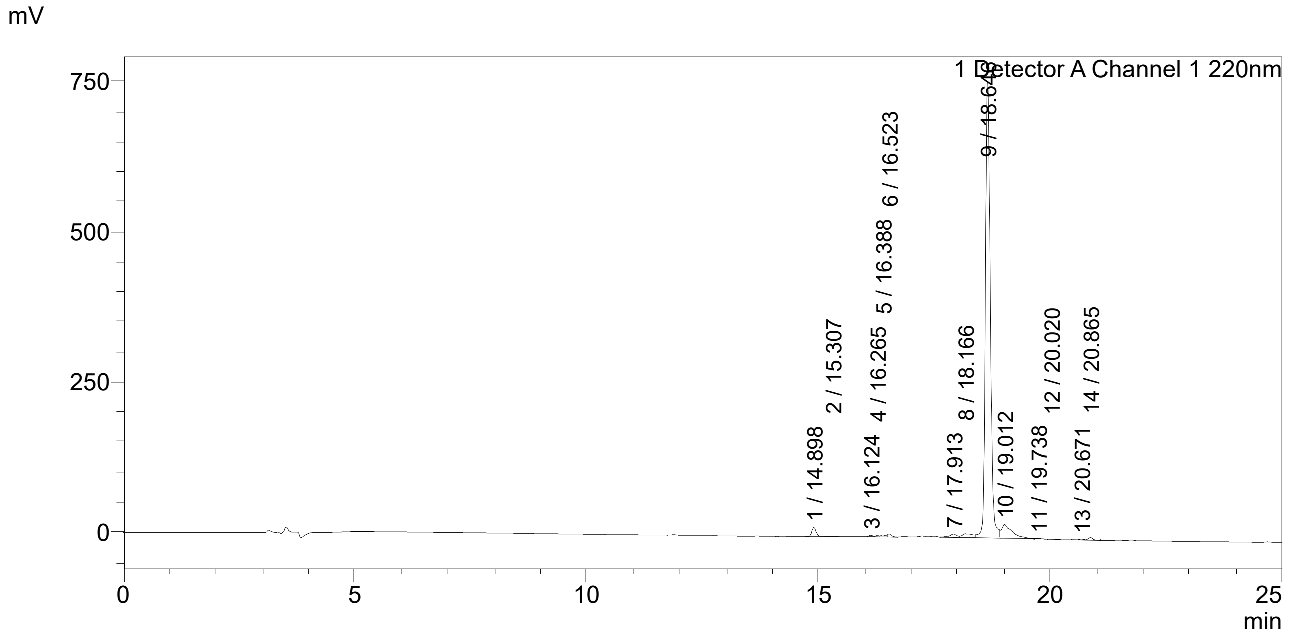

<Peak Table>

Detector A Channel 1 220nm

Peak#	Ret. Time	Area	Height	Area%
1	14.898	101465	15237	1.600
2	15.307	1458	243	0.023
3	16.124	13009	2192	0.205
4	16.265	10826	1923	0.171
5	16.388	24999	3010	0.394
6	16.523	27187	4826	0.429
7	17.913	60478	5386	0.953
8	18.166	95088	6172	1.499
9	18.646	5615644	759272	88.528
10	19.012	347975	22686	5.486
11	19.738	3828	521	0.060
12	20.020	3151	433	0.050
13	20.671	10037	1245	0.158
14	20.865	28191	4248	0.444
Total		6343336	827395	100.000

Mass Spectrum

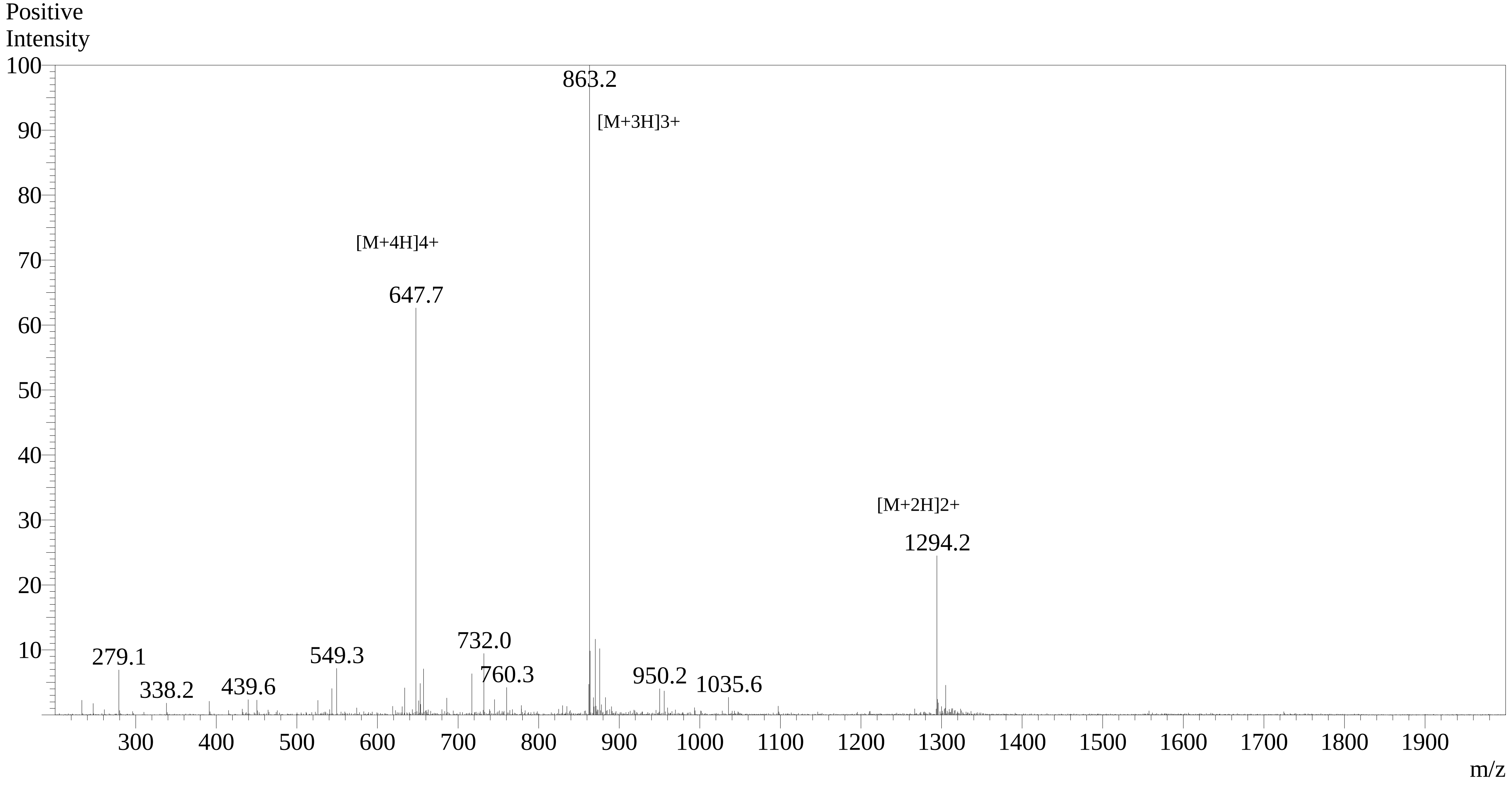

Sample Information	
Month-Day Processed :	11/13/20
Time Processed :	1:12:19
Injection Volume :	0.5
Sample Name :	NSP6_90-112
Sample ID :	U521TFI050-14
Theoretical MW :	2587.10
Observed MW :	2586.6

Interface	:ESI
Nebulizing Gas Flow	:1.5L/min
CDL Temp	:250
Block Temp	:200

Equipment	: GK11010007
Interface Bias	: +4.5 kV
Drying Gas Flow	:5 L/min
T.Flow	:0.2 ml/min
B.conc	:50%H2O/50%MeOH

Sample Name :NSP11_CoV1
Sample ID :U083MGG030-5
Time Processed :9:41:05 PM
Month-Day-Year Processed :08/26/2021

Pump A : 0.065% trifluoroacetic in 100% water (v/v)
Pump B : 0.05% trifluoroacetic in 100% acetonitrile (v/v)
Total Flow:1 ml/min
Wavelength:220 nm
<<LC Time Program>>

Time	Module	Command	Value
0.01	Pumps	B.Conc	5
25.00	Pumps	B.Conc	65
25.01	Pumps	B.Conc	95
27.00	Pumps	B.Conc	95
27.01	Pumps	B.Conc	5
35.00	Pumps	B.Conc	5
35.01	Controller	Stop	

<<Column Performance>>
<Detector A>
Column :Inertsil ODS-3 4.6 x 250 mm
Equipment: ZJ21010026

<Chromatogram>

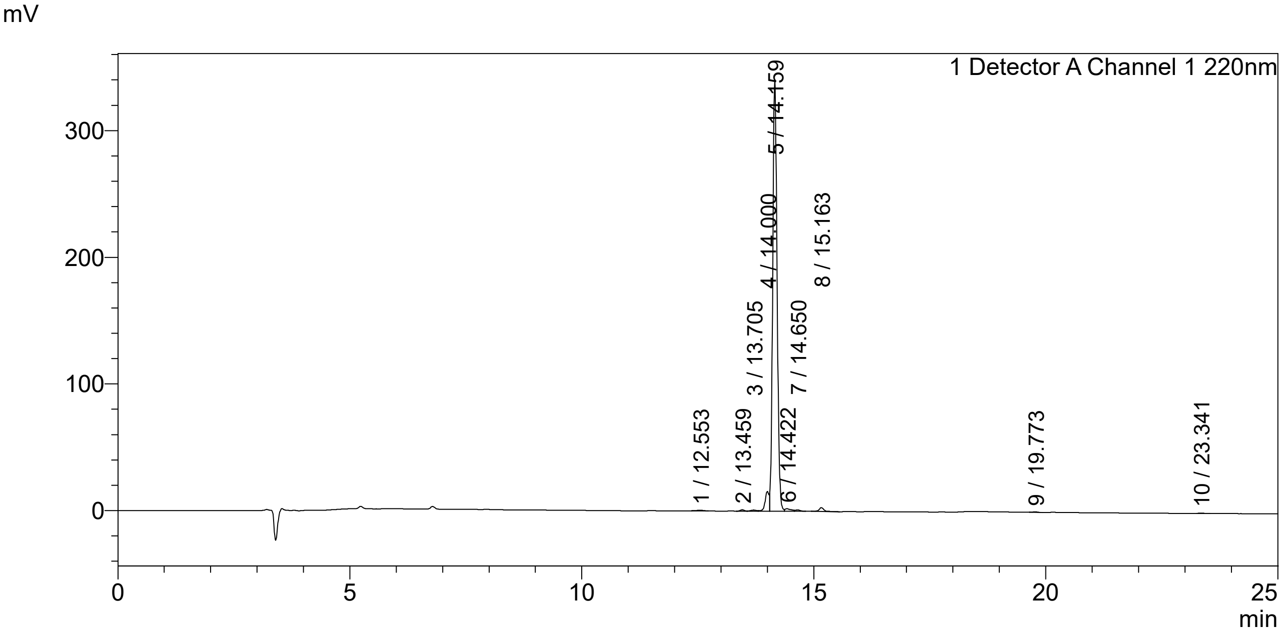

<Peak Table>

Detector A Channel 1 220nm

Peak#	Ret. Time	Area	Height	Area%
1	12.553	4698	491	0.195
2	13.459	6863	1130	0.285
3	13.705	7762	882	0.323
4	14.000	99865	15603	4.149
5	14.159	2235200	337815	92.872
6	14.422	18242	1969	0.758
7	14.650	7397	1061	0.307
8	15.163	22114	3009	0.919
9	19.773	2544	439	0.106
10	23.341	2070	319	0.086
Total		2406755	362718	100.000

Mass Spectrum

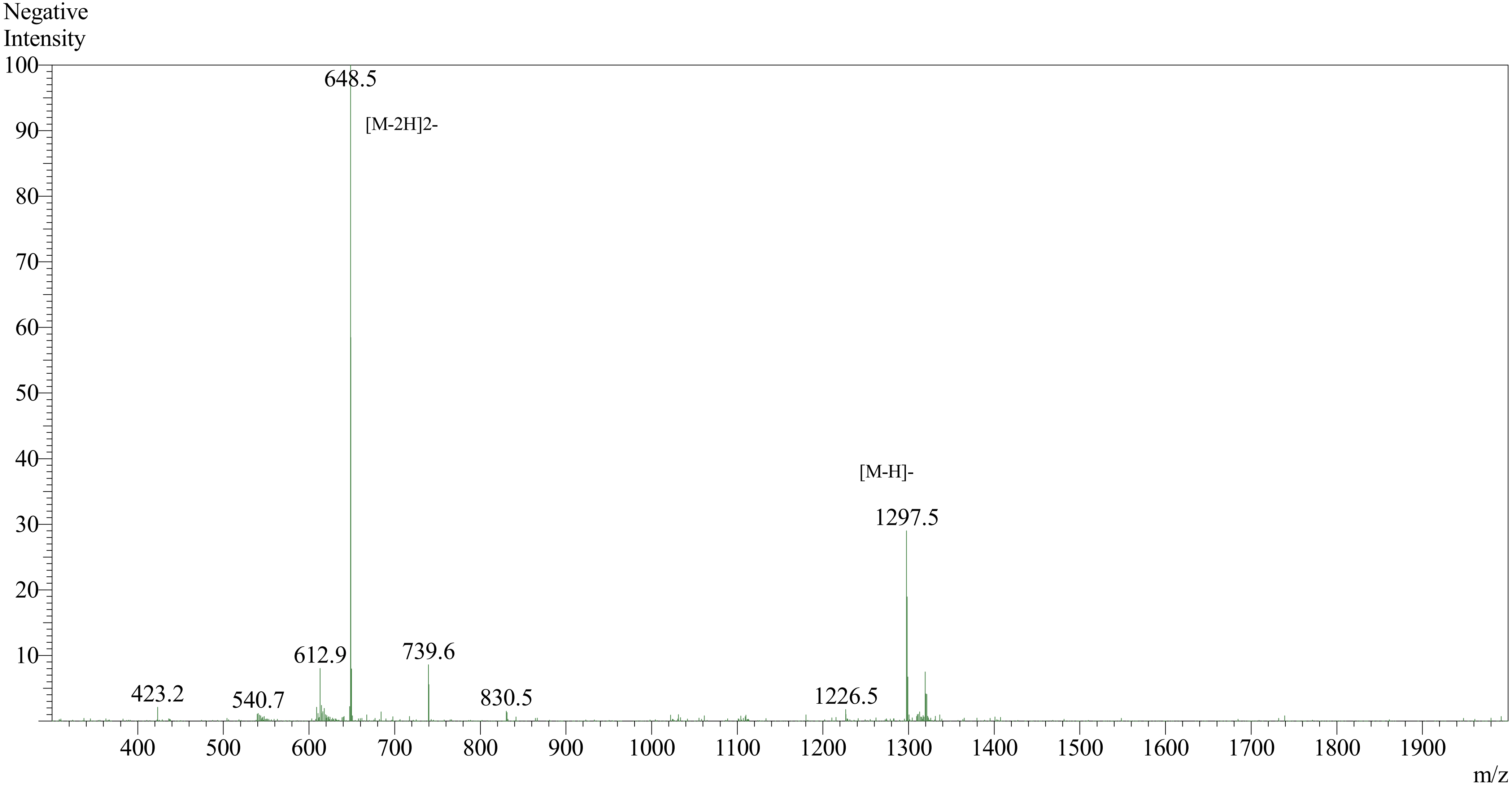

Sample Information	
Month-Day Processed :	08/26/21
Time Processed :	19:02:05
Injection Volume :	0.2
Sample Name :	NSP11_CoV1
Sample ID :	U083MGG030-5
Theoretical MW :	1299.39
Observed MW :	1299.0

Interface	:ESI
Nebulizing Gas Flow	:1.5L/min
CDL Temp	:250
Block Temp	:200

Equipment	: ZJ21010035
Interface Bias	: -3.5 kV
Drying Gas Flow	:5 L/min
T.Flow	:0.2 ml/min
B.conc	:50%H2O/50%MeOH

Sample Name: NSP11
Sample ID: U4944FD210-1
Time Processed : 22:23:11
Month-Day-Year Processed : 05/18/2020

Pump A : 0.065% trifluoroacetic in 100% water (v/v)
Pump B : 0.05% trifluoroacetic in 100% acetonitrile (v/v)
Total Flow:1 ml/min
Wavelength:220 nm
<<LC Time Program>>

Time	Module	Command	Value
0.01	Pumps	Pump B Conc.	5
25.00	Pumps	Pump B Conc.	65
25.01	Pumps	Pump B Conc.	95
27.00	Pumps	Pump B Conc.	95
27.01	Pumps	Pump B Conc.	5
32.00	Pumps	Pump B Conc.	5
32.01	Controller	Stop	

<<Column Performance>>
<Detector A>
Column :Inertsil ODS-3 4.6 x 250 mm
Equipment:GK12010012

<Chromatogram>

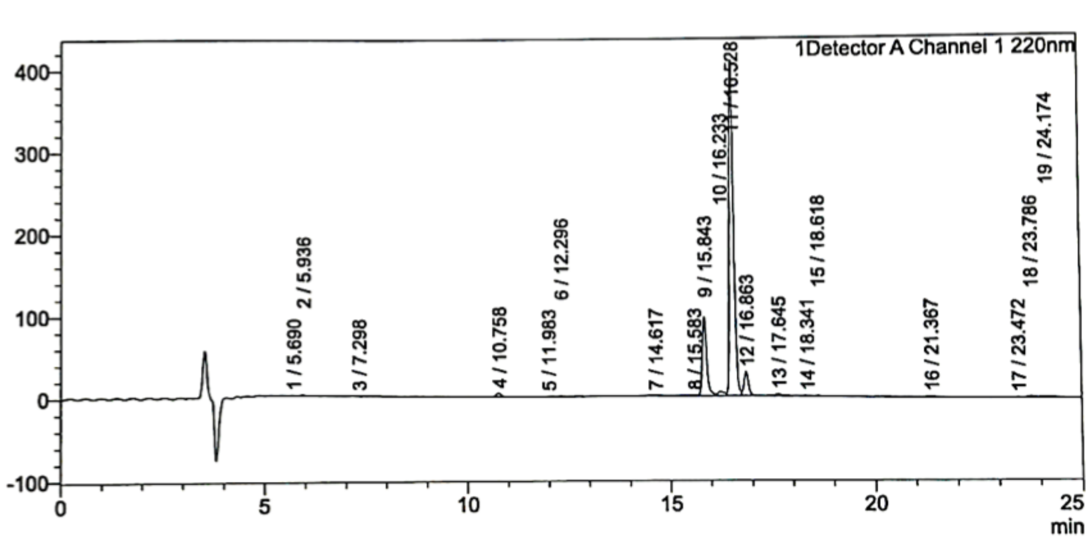

<Peak Table>

Detector A Channel 1 220nm

Peak#	Ret. Time	Area	Height	Area%
1	5.690	2349	339	0.056
2	5.936	4871	835	0.117
3	7.298	1508	363	0.036
4	10.758	31706	3942	0.763
5	11.983	4541	328	0.109
6	12.296	5427	502	0.131
7	14.617	4272	482	0.103
8	15.583	6546	657	0.157
9	15.843	704925	95447	16.955
10	16.233	54967	5099	1.322
11	16.528	3029858	408391	72.875
12	16.863	215824	28991	5.191
13	17.645	16702	2122	0.402
14	18.341	2876	443	0.069
15	18.618	5269	788	0.127

Peak#	Ret. Time	Area	Height	Area%
16	21.367	10405	999	0.250
17	23.472	4265	526	0.103
18	23.786	21649	1860	0.521
19	24.174	29680	1421	0.714
Total		4157638	553536	100.000

Mass Spectrum

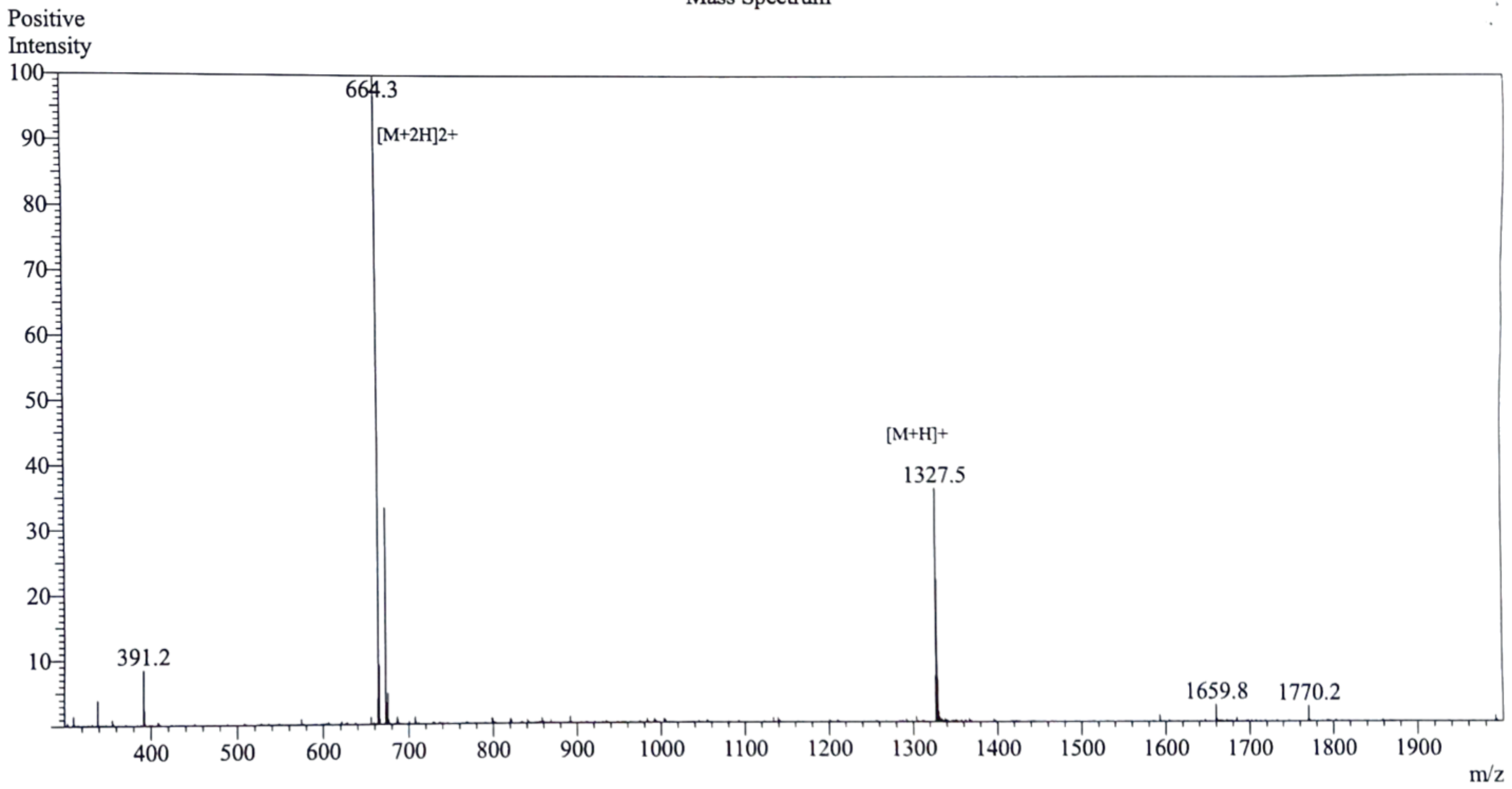

Sample Information	
Acquired by :	Gary
Month-Day Processed :	05/19/20
Time Processed :	06:59:54 AM
Injection Volume :	0.3
Sample Name :	NSP11
Sample ID :	U4944FD210-1
Theoretical MW :	1326.42
Observed MW :	1326.6

Interface	:ESI
Nebulizing Gas Flow	:1.5L/min
CDL Temp	:250
Block Temp	:200

Equipment	: GK11010007
Interface Bias	: +4.5 kV
Drying Gas Flow	:5 L/min
T.Flow	:0.2 ml/min
B.conc	:50%H2O/50%MeOH
