# Supplementary material for: Amyloidogenic proteins in the SARS-CoV and SARS-CoV-2 proteomes
Source: Nat Commun. 2023 Feb 20;14:945. doi: 10.1038/s41467-023-36234-4 (PMC9940680; doi:10.1038/s41467-023-36234-4)
Supplement: Supplementary file 5 — Reporting Summary [file 41467_2023_36234_MOESM5_ESM.pdf]

nature portfolio

Corresponding author(s): Dr. Rajanish Giri

Last updated by author(s): Jan 8, 2023

Reporting Summary

Nature Portfolio wishes to improve the reproducibility of the work that we publish. This form provides structure for consistency and transparency in reporting. For further information on Nature Portfolio policies, see our Editorial Policies and the Editorial Policy Checklist.

Statistics

For all statistical analyses, confirm that the following items are present in the figure legend, table legend, main text, or Methods section.

n/a	Confirmed	
☐	☒	The exact sample size (*n*) for each experimental group/condition, given as a discrete number and unit of measurement
☐	☒	A statement on whether measurements were taken from distinct samples or whether the same sample was measured repeatedly
☐	☒	The statistical test(s) used AND whether they are one- or two-sided<br>*Only common tests should be described solely by name; describe more complex techniques in the Methods section.*
☒	☐	A description of all covariates tested
☒	☐	A description of any assumptions or corrections, such as tests of normality and adjustment for multiple comparisons
☐	☒	A full description of the statistical parameters including central tendency (e.g. means) or other basic estimates (e.g. regression coefficient) AND variation (e.g. standard deviation) or associated estimates of uncertainty (e.g. confidence intervals)
☐	☒	For null hypothesis testing, the test statistic (e.g. *F*, *t*, *r*) with confidence intervals, effect sizes, degrees of freedom and *P* value noted<br>*Give P values as exact values whenever suitable.*
☒	☐	For Bayesian analysis, information on the choice of priors and Markov chain Monte Carlo settings
☒	☐	For hierarchical and complex designs, identification of the appropriate level for tests and full reporting of outcomes
☒	☐	Estimates of effect sizes (e.g. Cohen's *d*, Pearson's *r*), indicating how they were calculated

Our web collection on statistics for biologists contains articles on many of the points above.

Software and code

Policy information about availability of computer code

Data collection	MetAmyl (http://metamyl.genouest.org/e107_plugins/metamyl_aggregation/db_prediction_meta.php)<br>AGGRESCAN (http://bioinf.uab.es/aggrescan/)<br>FoldAmyloid (http://bioinfo.protres.ru/fold-amyloid/)<br>FISH Amyloid (https://www.ncbi.nlm.nih.gov/pmc/articles/PMC3941796/)<br>CamSol v2.2 10/2020 (http://www-vendruscolo.ch.cam.ac.uk/camsolmethod.html)<br>Origin Pro 2018 (9.5) (https://www.originlab.com/)<br>LabSpec 6 Spectroscopy Suite Software (https://www.horiba.com/int/scientific/products/detail/action/show/Product/labspec-6-spectroscopy-suite-software-1843/)<br>NetChop3.1 (https://services.healthtech.dtu.dk/service.php?NetChop-3.1)<br>GraphPad Prism 8.0.2 (https://www.graphpad.com/)
Data analysis	The bioinformatics prediction of all proteins of SARS-CoV and SARS-CoV-2 is done using MetAmyl, AGGRESCAN, FoldAmyloid, FISH Amyloid, and CamSol.<br>OriginLab and GraphPad Prism software are used to plot all data in the manuscript.

For manuscripts utilizing custom algorithms or software that are central to the research but not yet described in published literature, software must be made available to editors and reviewers. We strongly encourage code deposition in a community repository (e.g. GitHub). See the Nature Portfolio guidelines for submitting code & software for further information.

Data

Policy information about availability of data

All manuscripts must include a data availability statement. This statement should provide the following information, where applicable:

- Accession codes, unique identifiers, or web links for publicly available datasets
- A description of any restrictions on data availability
- For clinical datasets or third party data, please ensure that the statement adheres to our policy

All data are contained within the manuscript or as supporting information. Source data files associated with all the graphs are also provided with this paper. The sequences are retrieved from NCBI (https://www.ncbi.nlm.nih.gov/) and UniProt (https://www.uniprot.org/) data-bases. All the accession codes are provided in the respective sections of proteins in supplementary tables 1 to 6.

Human research participants

Policy information about studies involving human research participants and Sex and Gender in Research.

Reporting on sex and gender	This research article does not include any human research participants.
Population characteristics	Not applicable to this article.
Recruitment	Not applicable to this article.
Ethics oversight	Not applicable to this article.

Note that full information on the approval of the study protocol must also be provided in the manuscript.

Field-specific reporting

Please select the one below that is the best fit for your research. If you are not sure, read the appropriate sections before making your selection.

☒ Life sciences ☐ Behavioural & social sciences ☐ Ecological, evolutionary & environmental sciences

For a reference copy of the document with all sections, see nature.com/documents/nr-reporting-summary-flat.pdf

Life sciences study design

All studies must disclose on these points even when the disclosure is negative.

Sample size	We have used the SARS-CoV and SARS-CoV-2 proteins/regions for in-vitro aggregation using synthesized proteins. These peptides were selected based on the aggregation prone regions predicted by the web-servers. Here, a total number of 9 proteins and peptides are procured from Genescript (Spike signal and fusion peptides of both SARS-CoV and SARS-CoV-2, NSP1-p, NSP6-p, and NSP11 protein) and ThermoScientific Inc. (ORF10 protein) and validated for in-vitro aggregation.
Data exclusions	No data was excluded from the analysis.
Replication	Each experiment was successfully performed twice with two/three technical repeats. These replicates have produced similar results.
Randomization	We have used the synthesized peptides and performed aggregation related assays with biological and technical replicates. Among replicates, we have considered 2 or 3 technical replicates for calculating statistics therefore it does not require randomization in our data.
Blinding	Similar to above answer, among replicates, we have considered 2 or 3 technical replicates for calculating statistics therefore it does not require blinding either in our data.

Reporting for specific materials, systems and methods

We require information from authors about some types of materials, experimental systems and methods used in many studies. Here, indicate whether each material, system or method listed is relevant to your study. If you are not sure if a list item applies to your research, read the appropriate section before selecting a response.

Materials & experimental systems

n/a	Involved in the study
☒	☐ Antibodies
☐	☒ Eukaryotic cell lines
☒	☐ Palaeontology and archaeology
☒	☐ Animals and other organisms
☒	☐ Clinical data
☒	☐ Dual use research of concern

Methods

n/a	Involved in the study
☒	☐ ChIP-seq
☒	☐ Flow cytometry
☒	☐ MRI-based neuroimaging

Eukaryotic cell lines

Policy information about cell lines and Sex and Gender in Research

Cell line source(s)	SH-SY5Y neuroblastoma cells, HEPG2 hepatocellular carcinoma cells were procured from NCCS Pune, India (https://nccs.res.in/cellrepository).
Authentication	Both cell lines were authenticated through Short Tandem Repeat (STR) method. The STR loci were amplified using AmpFISTR Identifier Plus PCR Amplification Kit of Applied Bio systems. The cell line samples were processed using Applied Bio systems 3500 Genetic Analyzer. Data were analyzed using Gene Mapper ID-X5 v1.5 software. Both appropriate positive and negative were used and confirmed. For SH-SY5Y cells, percent match with ATCC STR profile database is 100% and for HepG2 cells percent match is 93%.
Mycoplasma contamination	Cell lines were not tested for Mycoplasma contamination.
Commonly misidentified lines (See ICLAC register)	HepG2 cells are contaminating cell lines as per ICLAC register.
